# The effect of manipulating glucuronic acid biosynthetic pathway in *Bacillus subtilis* strain on hyaluronic acid production

**DOI:** 10.1186/s13568-023-01567-2

**Published:** 2023-06-24

**Authors:** Shadi Afrasiabi, Fatemeh Sadat Amjad Zanjani, Gholamreza Ahmadian, Reza Ahangari Cohan, Malihe Keramati

**Affiliations:** 1 Department of Nanobiotechnology, New Technologies Research Group, Pasteur Institute of Iran, Tehran, Iran; 2 Department of Industrial and Environmental Biotechnology, National Institute for Genetic Engineering and Biotechnology (NIGEB), Tehran, Iran

**Keywords:** Biosynthetic pathway genes, Glucuronic acid, Hyaluronic acid, Metabolic engineering, Recombinant *Bacillus subtilis*

## Abstract

Hyaluronic acid (HA), composed of glucuronic acid (GlcUA) and N-acetyl glucoseamine (GlcNAc), is a versatile biopolymer with high commercial value and innumerous physiological roles and pharmaceutical applications. The *has*A gene has main role in HA biosynthesis by *Streptococcus* strain as a natural producer. The *has*B and *has*C genes are also mediate GlcUA precursor biosynthesis. In the present study, *S. equisimilis has*A gene; *B. subtilis tua*D and *gta*B genes for GlcUA precursors enhancement, and *vgb* gene coding bacterial hemoglobin as an oxygen provider were used to construct the *B*. *subtilis* strain for HA production. RBSHA (*has*A), RBSHA2 (*has*A/*tua*D/*gta*B), and RBSHA3 (*has*A/*tua*D/*gta*B/*vgb*) strains were developed and confirmed through genotype and phenotype analysis. After HA production and purification, FTIR spectroscopy confirmed the produced HA structures. HA assay showed the highest HA titer for RBSHA3 (2.1 ± 0.18 mg/ml) and then RBSHA2 (1.9 ± 0.03 mg/ml), and RBSHA (0.6 ± 0.14 mg/ml). Statistical analysis indicated there is no significant difference in HA titer between RBSHA2 and RBSHA3 strains (p-value > 0.05), however, these strains produced HA approximately 4-fold higher than that of RBSHA strain. Agarose gel electrophoresis showed the same molecular weight (< 30 kDa) of produced HA by strains. Dynamic light scattering (DLS) revealed all HA polymers had a relatively low polydispersity index (PDI < 0.5). These findings demonstrate the successful GlcUA biosynthetic pathway engineering strategy in improving HA yield by recombinant *B. subtilis*, metabolically-robust, and industrially potential strain.

**Supplementary Information:**

The online version contains supplementary material available at 10.1186/s13568-023-01567-2.

## Key points

• Overexpression of GlcUA biosynthesis genes improves HA titer up to 4-fold

• Engineered *B*. *subtilis* strains are capable of producing low PDI and low Mw HA

• The efficiency of VHb might not clearly detected in low viscose production media

**Supplementary Information:**

The online version contains supplementary material available at 10.1186/s13568-023-01567-2.

## Introduction

Hyaluronic acid (HA) is composed of disaccharide repeats of D-glucuronic acid (GlcUA) and N-acetylglucosamine (GlcNAc) joined alternately by b-1,3 and b-1,4 glycosidic bonds. This biopolymer is naturally produced by *Streptococcus* species including *S.zooepidemicus*, *S.equisimilis* group A and C, and *Pasteurella multocida* (Manfrão-Netto et al. 2022).

**Fig. 1:**
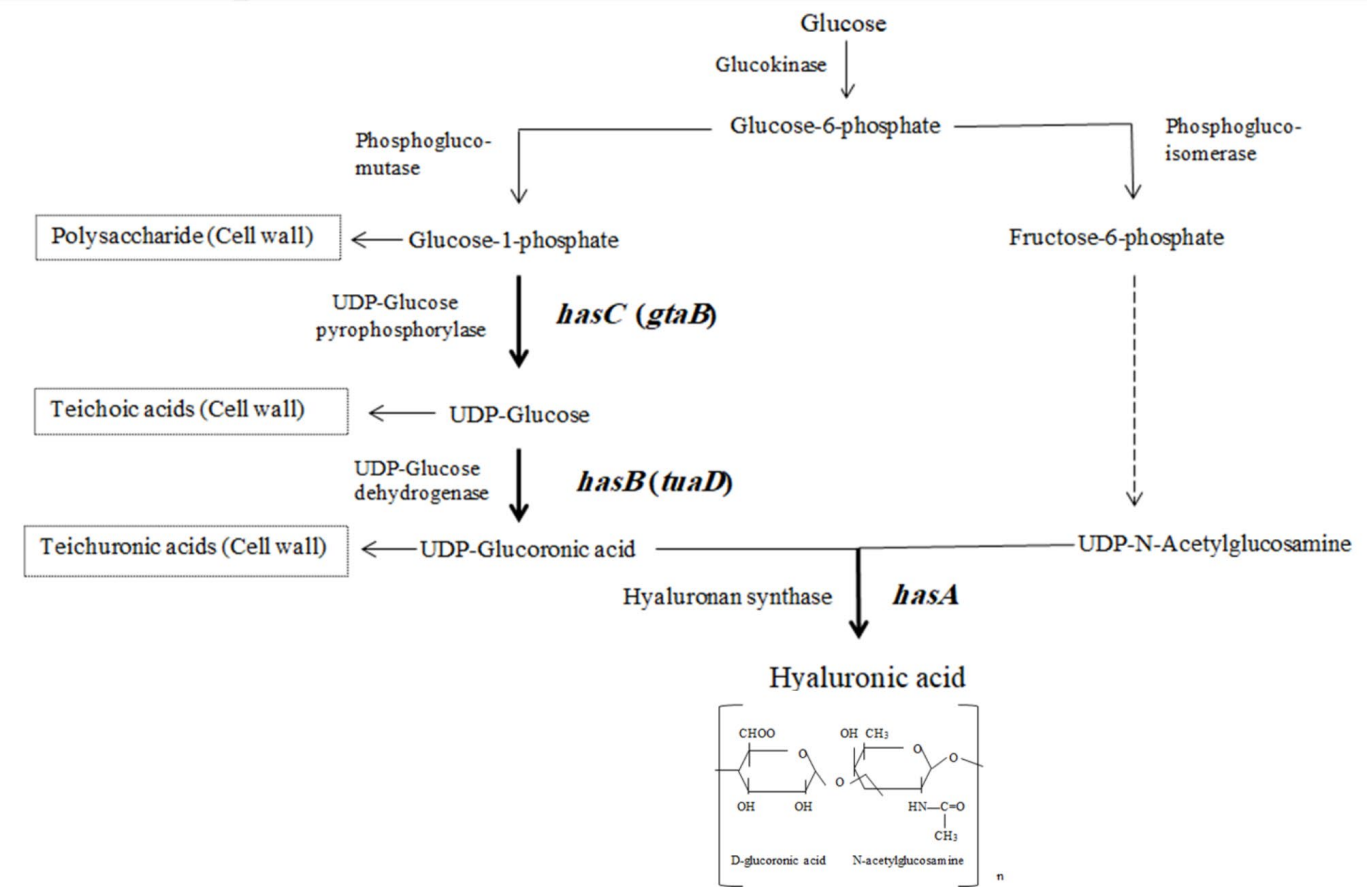
Hyaluronic acid biosynthesis pathway. Heterologous expressed genes in this study are *has*A, *tua*D, and *gta*B

Due to the distinctive properties of HA such as viscoelasticity, unique rheological feature, and moisturizing retention ability besides lack of immunogenicity and toxicity, it has found a wide variety of applications in the cosmetic, biomedical, and food industries (Bowman et al. 2018; Bukhari et al. 2018). Therefore, all related up and downstream processes for HA production including screening, selection, development of producer strains, optimization of production conditions, isolation and purification of the final product have been the main subjects of extensive studies from the past up to now (Manfrão-Netto et al. 2022; Rodriguez-Marquez et al. 2022). Although microbial fermentation is a desirable platform for HA production at industrial scale, however using *Streptococcal* resources encounter production expense issues such as the requirement of fastidious culture condition and safety concerns due to intrinsic exotoxins (Liu et al. 2011). The biosynthesis of HA in native producer microorganisms consists of two distinct metabolic pathways. Both pathways initiate with glucose 6-phosphate and end with two different nucleotide sugars of UDP-GlcUA and UDP-GlcNAc as substrates. Eventually, the hyaluronan synthase (HAS) enzyme catalyzes the substrates polymerization (Gunasekaran et al. 2020). In this regard, a preferable strategy for heterologous production of HA is using the microorganisms that have the precursor’s biosynthesis pathway. To complete the HA production pathway in these microorganisms, the required gene will be *has*A gene that encodes the HAS enzyme (Fig. 1). In the case of heterologous production of HA, there are several reports on cloning of *has*A gene form *Streptococcus* or *Pasteurella* in *Bacillus subtilis* (Cerminati 2021; Chien and Lee 2007; Jia et al. 2013; Jin et al. 2016; Li et al. 2019; Westbrook et al. 2018b; Widner et al. 2005).

The cloned *has*A gene is adequate for HA production in *B. subtilis* however, glucose 6-phosphate and also other precursor of GlcUA not only are used in HA biosynthesis but also, it is essential for important cellular functions such as bacterial growth and cell wall biosynthesis (Fig. 1). Consequently, for the cell to synthesize remarkable amounts of HA, it is imperative that these precursors should be available at adequate levels to balance both cell growth and HA biosynthesis. Therefore, along with the *has*A gene, overexpression of the genes involved in precursor biosynthesis seems necessary. It has shown that UDP-GlcUA precursor production is the bottleneck of the biosynthesis pathway (Gunasekaran et al. 2020; Widner et al. 2005). The *has*B and *has*C genes are involved in GlcUA biosynthesis pathway in *Streptococcus spp*. The counter endogenous genes of this critical precursor in *B*. *subtilis* are *tua*D and *gta*B, respectively (Fig. 1) (Manfrão-Netto et al. 2022). As a result, the overexpression of these endogenous genes in *B*. *subtilis* can potentially lead to the improvement of HA production.

HA biosynthesis in recombinant *B. subtilis* is a high-energy demand process. For every mole of HA unit, 2 moles of glucose, 5 moles of nucleoside triphosphates (3 as ATP and 2 as UTP), and one mole of acetyl-coenzyme A are consumed (Schiraldi et al. 2010b; Ucm et al. 2022). This could impose a substantial metabolic burden on the cell. *Vitreoscilla* is a gram-negative strict aerobic bacterium, harboring *vgb* gene coding *Vitreoscilla* hemoglobin (VHb) as an oxygen-binding protein to survive and grow in an O_2_-poor environment. Heterologous expression of VHb often enhances cell growth and product formation and improves the energy flow within the cell (Webster et al. 2021). On the other hand, increased broth viscosity due to the production of HA at high titers or high molecular weights (Mw) especially at the end of fermentation (Widner et al. 2005), leads to a decrease of dissolved oxygen (DO) level in the culture medium. Considering that lower DO is associated with reduced HA titer (Manfrão-Netto et al. 2022), the presence and expression of an oxygen-carrier protein, like VHb, may lead to an increase in HA production.

Considering the importance of the stable transcription of *has*A gene and overexpression of genes that mediate the biosynthesis of rate-limiting precursor GlcUA, in the present study, two different recombinant *B. subtilis* strains were developed. Both recombinant strains had an integrated *has*A gene from *S. equisimilis* at the *amy*E locus and *tua*D and *gta*B genes in a replicative plasmid (pDG148) for endogenous GlcUA precursor enhancement. In addition, one of them had also the *vgb* gene from *Vitreoscilla* sp that encodes the *Vitreoscilla* hemoglobin. The resulting plasmids are named pG2 harboring *tua*D/*gta*B and pG3 harboring *tua*D/*gta*B/*vgb* genes (Online Resource Fig. 2). Using the *has*A gene, which encodes the seHAS from *S. equisimilis* would take the advantage of high polymerization rate (> 9000 units per minute) (Gunasekaran et al. 2020). The proposed design provides the opportunity for assess the effect of VHb on HA production and development of novel producing strains. After polymer characterization by FTIR, agarose gel electrophoresis and DLS techniques were used to determine the molecular weight and polydispersity of polymer, respectively.

## Materials and methods

### Bacterial strains, plasmids, and culture conditions

The bacterial strains and plasmids that have been used or developed in this study were summarized in Table 1. The *B. subtilis* strain 168 was purchased from *Bacillus* Genetic Stock Centre (BGCS) and *E. coli* TOP10 cells were obtained from Invitrogen Company. The *E. coli* - *B. subtilis* shuttle vectors of pDHAFB was obtained from Novagen, Darmstadt and pDG148 was a gift from Ezio Riccac (Federico II University of Naples, Italy). All strains were cultured in LB medium containing appropriate antibiotics at 37 °C, 200 rpm except in the cases mentioned. Ampicillin (100 µg/mL), kanamycin (20 µg/mL), and chloramphenicol (5 µg/mL) were used as selection marker in *E. coli* and *B. subtilis* strains, respectively. The pDHAFB was used for the construction of the integrative construct of *has*A gene (pDHhas plasmid). The pDHAFB backbone, harboring *bla* gene coding β-lactamase and *cat* gene coding chloramphenicol acetyltransferase that are used as selection markers in *E. coli* and *B. subtilis* respectively. The vector also harbors *amyE*-F and *amyE*-B regions of amylase gene for integration at *amyE* locus of *B. subtilis* genome through a double-recombination event (Yamamoto et al. 2001). The pDG148 plasmid was used for the construction of the replicative construct containing *tua*D and *gta*B genes (pG2 plasmid) and *tua*D, *gta*B, and *vgb* genes (pG3 plasmid). For protein expression under the control of P_spac_ promoter, as an inducer-specific promoter, the bacterial culture was induced by isopropyl β-D-1-thiogalactopyranoside (IPTG) at the final concentration of 0.7 mM.

**Table 1:** Bacterial strains and plasmids used or developed in this study

Strain	Description	Reference
*E. coli* TOP10	Fˊ(*lacIq* Tn10 (*tetR*)) *mcrA*D Δ*lacX74 deoR nupG recA1 araD139*Δ(*ara-leu*)7697 *galU galK rpsL* (*strR*) *endA1*λ- propagation host	Invitrogen
*B. subtilis* 168	trpC2 (ATCC 33,712), (ATCC, Manassas, VA, USA)	(Harwood 1992)
RBSHA	*B. subtilis* 168 carrying pDHhas	This study
RBSHA2	*B. subtilis*168 carrying pDHhas and pG2	This study
RBSHA3	*B. subtilis* 168 carrying pDHhas and pG3	This study
**Plasmids**	**Description**	**Reference**
pDG148	Multicopy *B. subtilis* and *E. coli shuttle* vector; Ap^r^, Km^r^, replicative	(Stragier et al. 1988)
pG1	pDG148 carrying *has*A gene	This study
pG2	pDG148 carrying *tua*D and *gta*B genes	This study
pG3	pDG148 carrying *tua*D, *gta*B, and *vgb* genes	This study
pDHAFB	*B. subtilis* and E. *coli shuttle* vector; Ap^r^, amy E:[ Cm^r^, *lacZ*, Pspac]	(Yamamoto et al. 1999)
pDHhas	pDHAFB carrying *has*A gene	This study

### Integrative construct design

The coding sequence of *has*A gene from *S. equisimilis* (gi|225700893:201362–202,615, 1253 bp) was codon optimized, synthesized (Biomatik gene synthesis company, Canada), and primary cloned into pDG148 which was designated as pG1 plasmid (Online Resource Fig. 1b). The resulting optimized construct contains a *Hin*dIII restriction site at its 5´-end and an *Xba*I restriction site at the 3´-end. Also, it carries a 6-histidine tag followed by two stop codons and terminator sequence at the 3´-end of the construct to enhance the mRNA stability (Online Resource Fig. 1a). The construct was then subcloned into an integrative pDHAFB vector (Online Resource Fig. 3) which harbors P_spac_ promoter to drive the expression of *has*A gene in *B. subtilis*. The resulting recombinant vector which is further designated as pDHhas, would be able to integrate the *has*A gene at the chromosomal locus of the amylase gene of *B. subtilis*.

### The construction and verification of pDHhas

Due to the low transformation rate of monomeric forms of plasmids into *B*. *subtilis* (Westbrook et al. 2016), along with *E. coli* Top 10, the propagation of recombinant plasmids was performed in *E. coli* C600 to obtain both monomer (mopDHhas) and multimeric (mupDHhas) forms of the plasmids, respectively (Voß et al. 2003). The *has*A gene was cloned at *Hin*dIII and *Xba*I sites of pDHAFB plasmid. The recombinant pDHhas was confirmed by sequencing of cloned *has*A gene using hasA-F, hasA-IF, and hasA-R primers (Online Resource Table 1) and restriction map analysis (Online Resource Fig. 1C). The monomeric (mopDHhas) and multimeric (mupDHhas) forms of plasmid were extracted from *E. coli* Top 10 and *E. coli* C600, respectively.

### Design and construction of replicative constructs

The replicative construct containing *tua*D (number: 749,168,884, 1383 bp) and *gta*B (gi number: 255,767,013, 876 bp) genes from *B. subtilis* 168 and *vgb* gene (gi number: AF292694.1, 438 bp) from *Vitreoscilla* was designed (Online Resource Fig. 2a) with optimization. The gene construct sequences were deposited to GenBank database. The genes were inserted between *Mau*BI and *Bam*HI restriction sites of pDG148 vector which further designated as pG3 (Online Resource Fig. 2b). The construct utilizes an optimized PliaI promoter (P_*lia*I(opt)_) (Toymentseva et al. 2012), a strong endogenous ribosome binding site, and a spacer at the upstream, a 6x-histidine tag, and a stop codon at the downstream of each gene. In addition, to enhance the mRNA stability a strong transcription terminator from the *Tgyr* gene of *B. subtilis* was placed at the 3´-end of the construct. The *Xho*I restriction site was located at either side of the *vgb* gene for the possibility of *vgb* (480 bp fragment) removal from pG3 plasmid to construct pG2 plasmid containing just *tua*D and *gta*B genes (Online Resource Fig. 2c and Fig. 6). The monomer (mopG2) and multimeric (mupG2) forms of PG2 were respectively extracted from propagation hosts, E. *coli* Top10 and E. *coli* C600 strains, and confirmed by sequencing and restriction map analysis.

### *B. subtilis* competent cell preparation and transformation of pDHhas

Transformation of *B. subtilis* strain was performed using a conventional protocol developed by Anagnostopoulos and Spizizen for natural competence with some modifications (Anagnostopoulos and Spizizen 1961). The pre-culture of *B. subtilis* was carried out at 37 °C and 180 rpm for 18 h. The cells were then plated on a non-select LB agar and incubated overnight. Five microliters LB medium was inoculated by a single clone of *B. subtilis* and incubated for another 18 h as the seed culture. The seed culture was then diluted 50-fold in fresh LB medium and incubated at 200 rpm to OD_600_ ~ 0.3. Then, 30 ml cultured cells were harvested and re-suspended in 10 ml SP1 medium (Table 2) and incubated at 60 rpm to OD_600_ 2.6. The SP2 medium (Table 2) was inoculated by SP1 with a ration of 1:10 and incubated at 40 rpm for 2 h. The prepared competent cells were finally transformed by 1 µg pDHhas plasmid and incubated at 40 rpm for another 2 h. The cell suspension was plated on LB agar supplemented with chloramphenicol and incubated for 20 h to select the recombinant clones of *B. subtilis* harboring pDHhas (RBSHA).

**Table 2:** The ingredients of SP1 and SP2 media

Media	Ingredients
SP1	Glucose 0.5% + MgSO_4_ -7H_2_O 0.02%, Casamino acid 0.02%, Tryptophan 0.005%, (NH_4_)_2_SO_4_ 0.2%, K_2_HPO_4_ 1.4%, KH_2_PO_4_ 0.6%, Na_3_C_6_H_5_O_7_-2H_2_O 0.1%
SP2	Glucose 0.45% + MgSO_4_ -7H_2_O 0.018%, Casamino acid 0.009%, Tryptophan 0.00045%, (NH_4_)_2_SO_4_ 0.18%, K_2_HPO_4_ 1.26%, KH_2_PO_4_ 0.54%, Na_3_C_6_H_5_O_7_-2H_2_O 0.09%

### Phenotype and genotype analysis of recombinant *B. subtilis*

The wild type *B. subtilis* produces α-amylase enzyme on starch agar that creates a clear zone around the colonies after iodine staining due to digestion of starch. Therefore, if the α-amylase coding gene is disrupted by an external gene, the clear zone cannot be formed. In this regard, the RBSHA cells were grown on nutrient agar plate supplemented with 1% soluble starch approximately for 20 h at 37 °C. To determine the clear zone, the surface of the media was flooded with an iodine staining solution (0.5% iodine and 1% potassium iodide) (Lal and Cheeptham 2012). The colonies without clear zone were considered amylase negative and presumed *amy*E knockouts meant the successful integration of the *has*A gene into the genome. The selected clones were analyzed for the presence of *has*A gene by PCR. In brief, genomic DNA was extracted using HiGene™ genomic DNA prep kit (BioFact, Korea) and used as the template for *has*A gene amplification by designed primers (hasA-F:TCCAGAACAACCTCTGCTAAAATTCC, hasA-IF: TCTAATGTTATCGTTCATCGCTCAG, and hasA-R: CGAGGTCATCATTTCCTTCCGAA, Online Resource Table 1). the PCR program was as follows; 95 °C for 60 s, 63.8 °C for 60 s, and 72 °C for 80 s. The PCR products were analyzed on 1% agarose gel electrophoresis.

### Development and confirmation of RBSHA2 and RBSHA3 strains

The RBSHA competent cells harboring the integrated *has*A gene were prepared as described previously and transformed by replicative pG2 or pG3 plasmids to form RBSHA2 or RBSHA3, respectively. To increase transformation efficiency, both monomer and multimeric forms of each plasmid (pG2 and pG3) were employed. Finally, the transformed cells were plated on LB agar supplemented with two selection markers (chloramphenicol and kanamycin) and incubated for 16 h for further screening and selection. The pG2 and pG3 plasmids were extracted from an overnight culture of RBSHA2 and RBSHA3 strains using a miniprep plasmid extraction kit (Favorgene, Taiwan), and the accuracy of the cloning were analyzed by restriction map analysis and sequencing.

### HAS expression in recombinant RBSHA strain

To investigate HAS expression, 5 ml LB medium was inoculated with an overnight culture of RBSHA and incubated for 14 h at 37 °C / 150 rpm as the seed culture. The seed culture (4 ml) was transferred into 120 ml LB to OD_600_ ~ 0.3, in which the P_spac_ promoter was induced by IPTG at a final concentration of 1 mM. The induced culture was incubated at 37 °C and 150 rpm for an overnight. The cultured cells were harvested by centrifugation at 6000 g (4 °C) and washed with PBS three times. For HAS extraction, the cell pellet was re-suspended in 2 ml lysis buffer containing 50 mM NaH_2_PO_4_, 300 mM NaCl, 10 mM imidazole, and lysozyme 2.5 mg/ml. The lysate was incubated for 30 min on ice and sonicated (Ultrasonic liquid processor, BANDELIN, German) intermittently (7 pulses; each included: 10” burst, 10” cooling). Afterward, RNaseA (10 µg/ml) and DNase (5 µg/ml) were added and mixed thoroughly. The prepared lysate was centrifuged at 10,000 g, 4ºC for 30 min. The protein purification was performed under native conditions using Ni-NTA resins (QIAGEN, Germany) according to the manufacturer’s protocol. The purified proteins were analyzed by SDS-PAGE technique (Green et al. 2012).

### Protein expression in RBSHA2 and RBSHA3 strains

The expression of TuaD and GtaB in RBSHA2 and TuaD, GtaB, and VHb in RBSHA3 were performed similarly to the expression HAS in RBSHA as previously described. The cell lysates were then examined for recombinant protein expression by standard SDS-PAGE and Western blot procedures.

### HA production, purification, and titration

Screening of recombinant strains was performed to evaluate HA production. For this purpose, several colonies of RBSHA, RBSHA2, and RBSHA3 strains were plated on LB agar media and grown overnight at 37ºC. The cultured cells were used to inoculate 5 ml LB broth and incubated for 16 h at 37ºC and 150 rpm as starter cultures. The starter was then used to inoculate 10 ml LB media (3% v/v) and incubated at 37ºC and 180 rpm to OD_600_ ~ 0.3. The cultured cells were further induced by IPTG (0.7 mM). After 20 h incubation at 37ºC and 150 rpm, glucose at a final concentration of 2% was added and the incubation was continued for another 5 h under the same conditions. The cultured cells were removed by centrifugation and the supernatant was subjected to purification step. HA purification was performed in five steps as described previously with minor modifications (Amjad Zanjani et al. 2022). (1) Precipitation of protein impurities was done by the addition of trichloroacetic acid (TCA, 100% w/v) and incubation at 4ºC for 1.5 h followed by centrifugation at 5000 g for 20 min at 4ºC. (2) Further reduction in protein impurity was carried out by sodium dodecyl sulfate (1% w/v) treatment for 15 min at 37 °C, 200 rpm. (3) Conversion of HA to sodium hyaluronate salt was obtained by adding NaCl (0.5 M). (4) HA precipitation was achieved by adding absolute ethanol (2:1 v/v) and incubation at 4ºC for 16 h. (5) The removal of DNA impurities was performed by activated charcoal (1% w/v) treatment followed by centrifugation. Finally, the purified HAs were dissolved in NaCl 0.15 M and analyzed for determination of DNA and protein impurities via UV-spectroscopy (Thermo, USA). The HA concentration in the samples was measured by carbazole assay according to Cesaretti et al. (Cesaretti et al. 2003). A serial dilution of glucuronic acid solution (0 to 1000 µg/ml) was used for standard curve plotting. All experiments were performed in triplicate. A 10 kDa HA standard (Bloomage Biotechnology Corp.) was used as control. The significance difference between HA titers among recombinant strains was statistically determined using one-way ANOVA test (p-value < 0.05, GraphPad Prism software).

### HA characterization

The Mw of HA was analyzed via agarose gel electrophoresis. Samples containing 6 µg purified HA and low molecular HA ladder (Echelon Biosciences Inc., USA) were separately mixed with 30% glycerol and loaded on the wells. Agarose electrophoresis was carried out at room temperature for 1 h with a constant voltage of 100 V. After running, the gel was placed in ethanol 50% for 1 h, and in 0.005% Stains-all® staining solution (dissolved in 50% v/v in ethanol) for 18 h in light-protective conditions. De-staining was done by photo bleaching in front of the light till the HA bands appeared. Also, the agarose gel was analyzed by gel analyzer software (Gel Analyzer version 19.1). In addition, the polydispersity index (PDI) of HA was determined using a dynamic light scattering (DLS) technique (Malvern Instrument, U.K). The identity of purified HAs was confirmed by FTIR analysis. For this purpose, the samples and control were dried and analyzed by spectroscopy apparatus (Thermo, USA) at a range of 500–4000 cm^− 1^.

## Results

### Verification of gene constructs

The gene constructs containing the *has*A and *tua*D, *gta*B and *vgb* received the accession No: LC753992 and LC753993, respectively. Restriction enzyme analysis of pDHhas plasmid from both propagation hosts (*E. coli* Top10 and *E. coli* C600 strains) confirmed the insertion of *has*A gene. A 1386 bp fragment of *has*A gene in the examined clones is shown in (Online Resource Fig. 1C). As well as pDHhas, the replicative plasmids including pG2 and pG3, respectively harboring *gta*B/*tua*D and *gta*B/*tua*D /*vgb*, genes were extracted from both propagation hosts. The pG3 restriction map analysis was performed by 1% agarose gel electrophoresis. *Xho*I digested mopG3 and mupG3 represented two distinct bands including a linearized pG3 band at 9389 bp and a 480 bp band related to the *vgb* gene (Online Resource Fig. 5a and b, and 5c). In addition, pG2 restriction map analysis showed that the *Xho*I digestion resulted in a single band at 9389 bp for both mupG2 and mopG2, which in turn confirms the clones (Online Resource Fig. 7a and b).

### Phenotype and genotype analysis of RBSHA strains

**Fig. 2:**
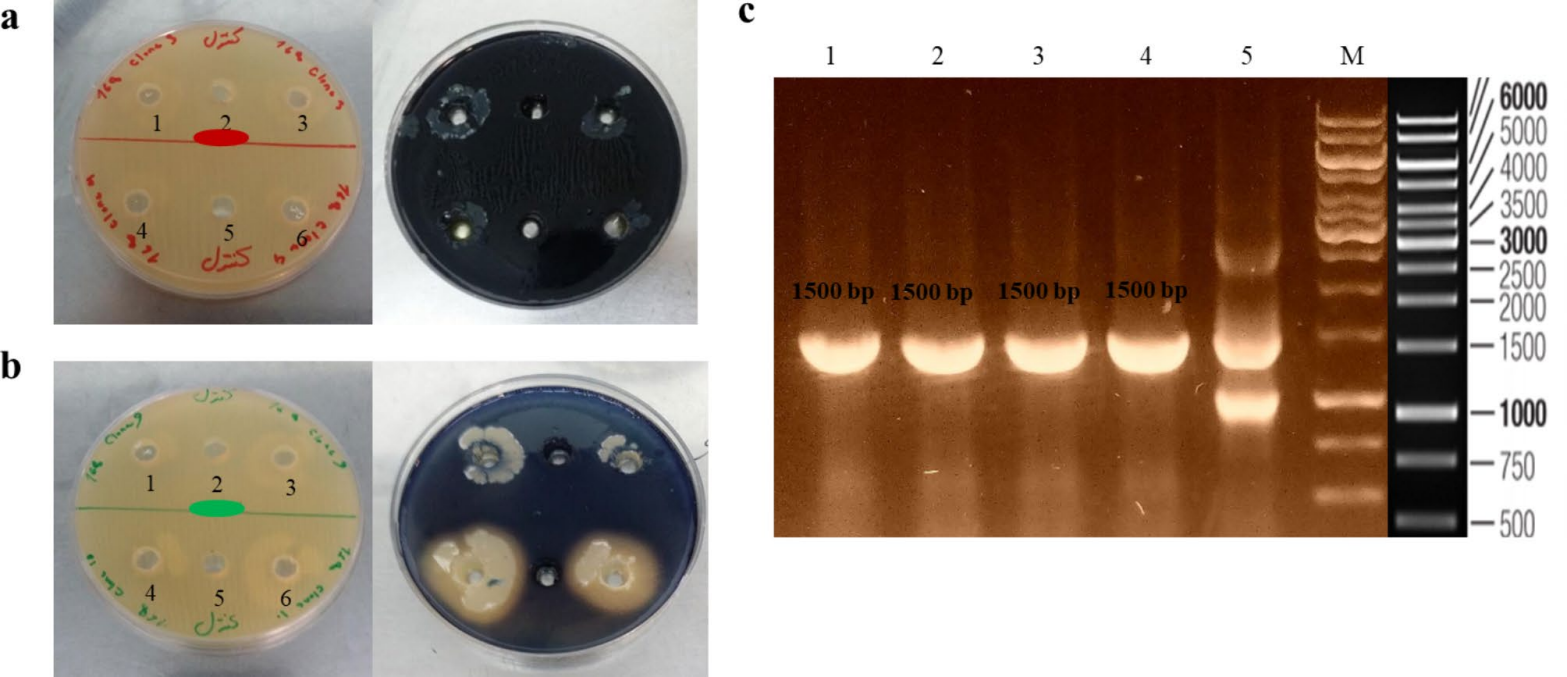
Phenotypic and genotypic study of pDHhas integration in the genome of *B. subtilis*. **(a)** Amylase function study in the recombinant *B. subtilis*. Wells 1, 3, 4, and 6 are recombinant clone harboring pDHhas and wells 2 and 5 are LB broth medium minus bacteria before (left) and after (right) pouring iodine solution. **(b)** Amylase function study in recombinant *B. subtilis*. Lanes 1, 3, 4, and 6 are recombinant clones harboring pDHhas and lanes 2 and 5 are LB broth medium minus bacteria before (left) and after (right) pouring iodine solution. **(c)** Agarose gel electrophoresis of *has*A PCR product from RBSHA genome [Lanes 1 to 4: PCR product of four RBSHA clones with hasA-F and hasA-R primers, confirmed by phenotypic test; lane 5: PCR product of RBSHA clone with hasA-F, hasA-IF, and hasA-R primers, confirmed by phenotypic test; M: 1 kb DNA size marker]. PCR amplified *has*A genes with the expected length of 1500 bp are shown in lanes 1, 2, 3, and 4

Based on the amylase phenotypic test, the lack of clear zone around the wells indicated the disruption of α-amylase gene by the *has*A insertion (Fig. 2a and b). The verified clones with the disrupted α-amylase gene were designated as RBSHA strains. Also, genetic analysis was performed using PCR test that covered the entire length of the *hasA* gene. The amplified product with an expected length (1500 bp) confirmed the integration of *has*A gene in the chromosome of all tested RBSHA strains (Fig. 2c).

### Genotype analysis of RBSHA2 and RBSHA3 strains

The pG2 and pG3 plasmids, respectively extracted from RBSAH2 and RBSH3 strains, were analyzed by restriction enzyme analysis. The pG3 plasmid has three cleavage sites for *Xba*I and each of them is located in the spacer sequence at the beginning of *tua*D, *gta*B, and *vgb* genes. Accordingly, in plasmid PG2 which lacks *vgb* gene, there are only two cleavage sites for this restriction enzyme (Online Resource Fig. 2d). As expected, two bands at 1419 and 7970 bp were achieved for pG2 digestion which related to the sequence between *tua*D and *gta*B spacer and the sequence out of *tua*D and *gta*B spacer, respectively. Similarly, three bands at 918, 1419 and 7532 bp correspond to the sequence between *gta*B and *vgb* spacer, the sequence between *tua*D and *gta*B spacer, and the sequence out of *vgb* and *tua*D spacer for pG3 digestion (Online Resource Fig. 8).

### Expression analysis in recombinant RBSHA, RBSHA2, and RBSHA3 strains

**Fig. 3:**
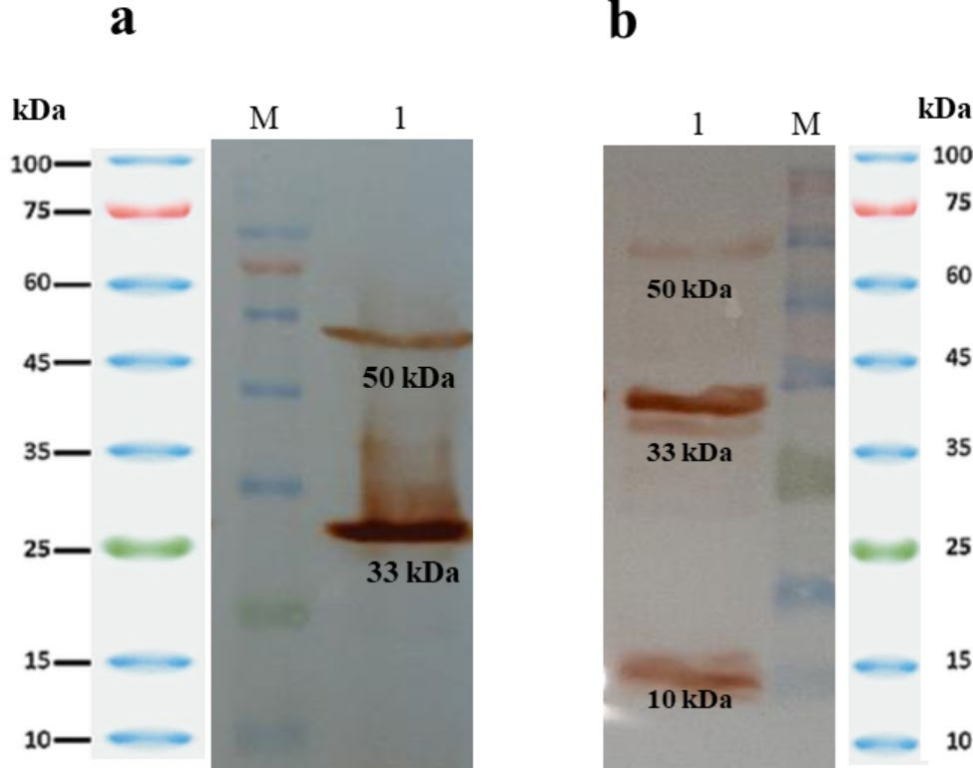
Protein identification in RBSHA2 and RBSHA3 strains by western blot analysis on cell extracts. **(a)** Protein identification of RBSHA2 strain. [M: protein marker; lane 1: cell extract of RBSHA2]. TuaD and GtaB expressed proteins are seen at 50 kDa and 33 kDa in lane 1. **(b)** Protein identification of RBSHA3 strain. Lane 1: cell extract of RBSHA3; M: protein marker]. TuaD, GtaB, and VHb expressed proteins are seen at 50 kDa, 33 kDa, and 10 kDa in lane 1

The expression of HAS enzyme was successfully verified in the RBSHA strains. The corresponding band had a molecular weight of ~ 49 kDa (Online Resource Fig. 9). In addition, western blot analysis showed the expression of TuaD and GtaB at 50 and 33 kDa in RBSHA2 and also, TuaD, GtaB, and VHb at 50, 33, and 10 kDa in RBSHA3, respectively (Fig. 3).

### HA purification, quantification, and characterization

**Fig. 4:**
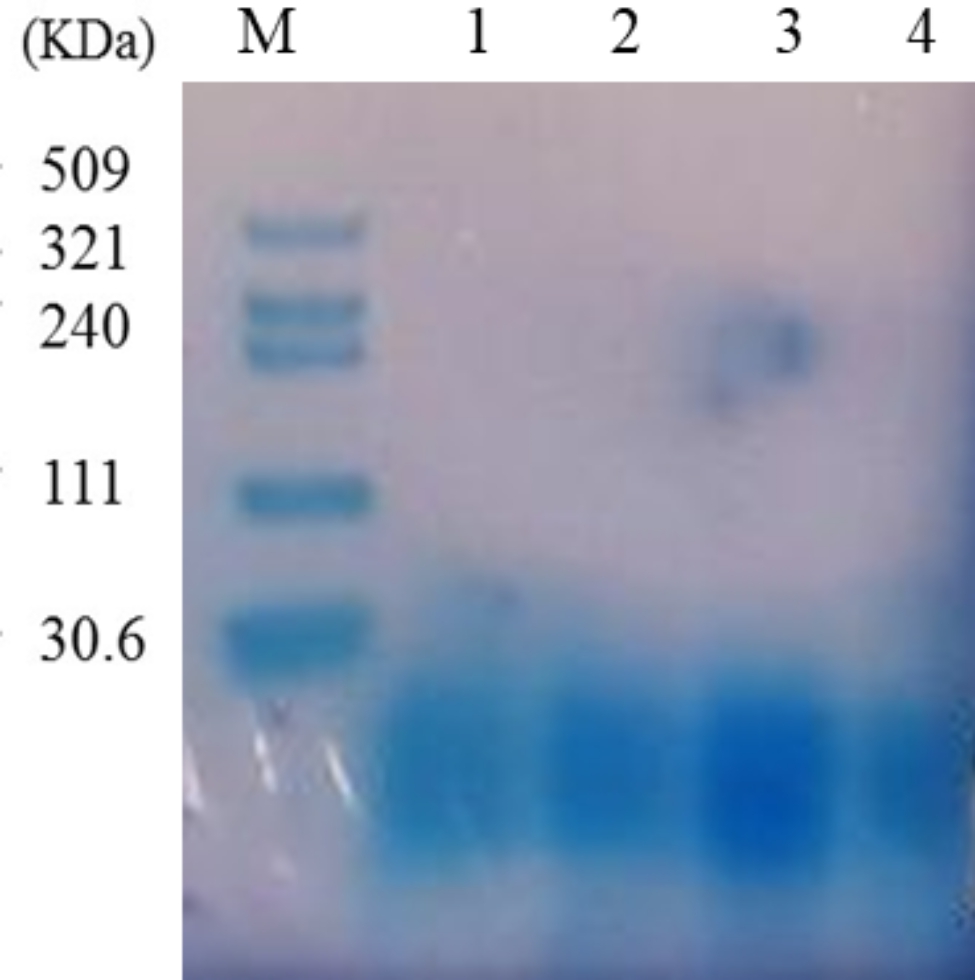
Agarose gel electrophoresis of purified HAs from RBSHA2 and RBSHA3 strains using Stains-all® method. [M: HA ladder; Lane 1: the purified HA produced by RBSHA3, lane 2: the purified HA produced by RBSHA2; lane 3: purified HA produced by RBSHA2 and lane 4: purified HA produced by RBSHA3]. As seen in lanes 1, 2, 3, and 4, all purified HAs from RBSHA2 and RBSHA3 strains show the same Mw of HA that is less than 30 kDa

**Fig. 5:**
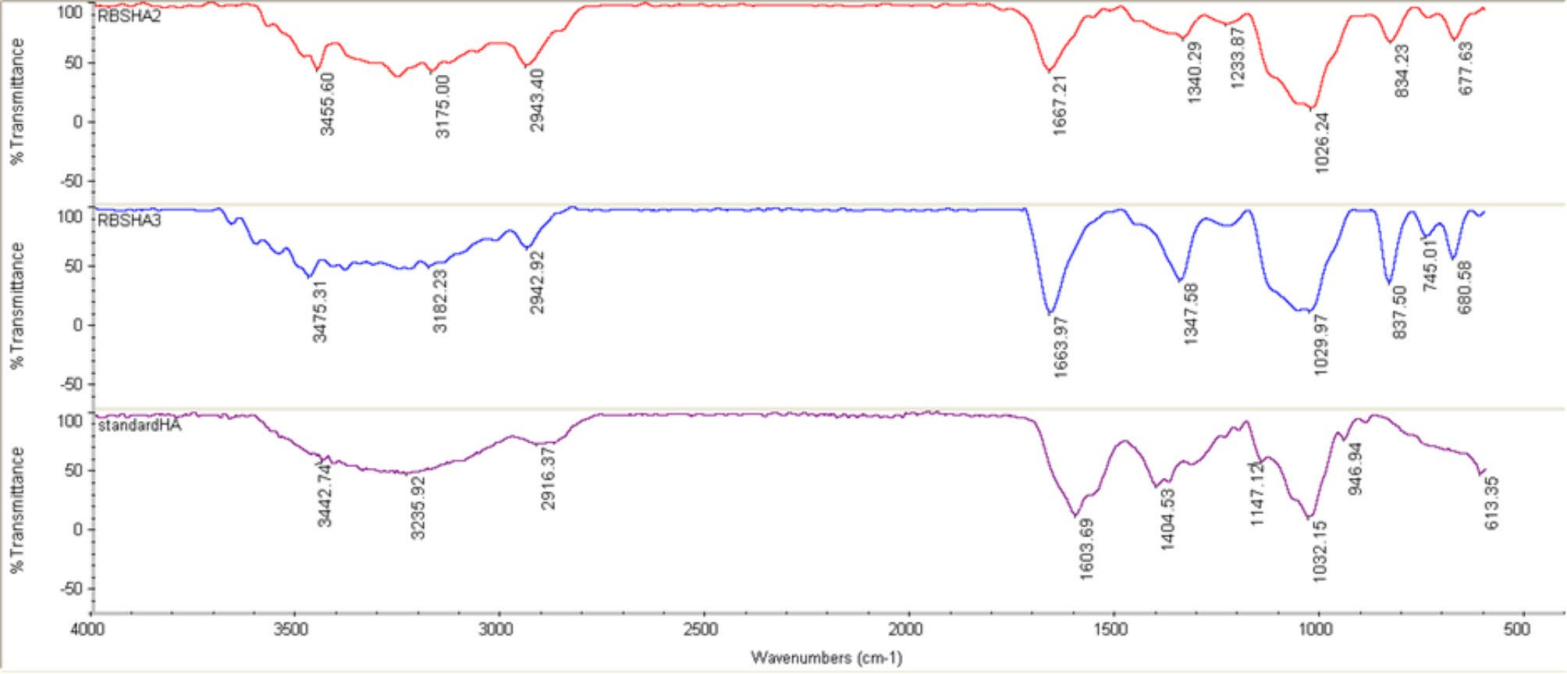
FTIR spectra of purified HAs from RBSHA2 and RBSHA3 strains along with the standard. The purple graph: standard HA. The blue graph: purified HA from RBSHA3 strain, and the red graph: purified HA from RBSHA2 strain

Within the hyaluronic acid purification procedure, we employed TCA treatment as the first step. TCA-induced protein precipitation is independent of protein size and nature (Rajalingam et al. 2009), allowing it to effectively precipitate a wide range of unwanted proteins from our culture broth and significantly reduce the protein load. This results in a limited amount of protein remaining to be co-precipitated with hyaluronic acid in subsequent purification steps. Furthermore, TCA addition to the fermentation broth not only causes protein precipitation but also promotes flocculation, making cell separation easier and reducing viscosity. These desirable effects contribute to safe, fast, and effective clarification of the broth (Schiraldi et al. 2010a). In addition to TCA treatment, we utilized activated carbon treatment to remove high molecular weight remaining protein impurities. The combination of these two purification steps resulted in a significant reduction in the protein load. Before HA titer, DNA and protein impurities were determined by optical density measurements at 260 and 280 nm wavelengths, respectively. The findings showed that impurities were in the range of acceptance limit according to the HA monograph of European Pharmacopoeia 10 (Online Resource Table 2). HA titration assay was performed for recombinant RBSHA, RSHA2, and RBSHA3 strains. Based on the carbazole assay, HA production by RBSHA was in a range of 0.08 to 0.55 mg/ml which only carries *has*A gene. Meanwhile, HA titer for RBSHA2 harboring *has*A, *tua*D, and *gta*B genes ranged from 1.21 to 1.90 mg/ml. and the HA titer for RBSHA3 harboring *has*A, *tua*D, *gta*B, and *vgb* genes was from 1.37 to 2.06 mg/ml (Table 3). Molecular weight analysis of purified HAs from RBSHA2 and RBSHA3 strains revealed these recombinant strains produce the same Mw of HA (< 30 kDa) (Fig. 4) which is considered as low Mw HA (Radrezza et al. 2020). The PDI of all HA samples was lower than 0.5, indicating the low polydispersity of the produced HA molecules. The FTIR spectra of purified HAs from recombinant strains showed the same pattern in comparison with the standard (Fig. 5). The absorption peak at 2916 cm^− 1^ can be related to CH symmetrical and CH2 asymmetrical stretching. The peaks at positions 1603 cm^− 1^, 1404 cm^− 1^, and 1147 cm^− 1^ can be attributed to amides I, II, and III. The absorption peak at 1032 cm^− 1^ can be related to the carbohydrate molecule (Chen et al. 2019; Gilli et al. 1994).

**Table 3:** The HA production by the recombinant *B. subtilis* strains

Recombinant strain	Clone No	STD	RSD (%)	HA titer (mg/ml)
RBSHA	C1	0.11	5.87	0.08
	C2	0.10	5.95	0.32
	C3	0.01	0.74	0.18
	C4	0.09	5.99	0.23
	C5	0.16	7.06	0.21
	C6	0.14	5.94	0.55
	C7	0.11	6.00	0.35
RBSHA2	C_2g_1	0.18	9.72	1.57
	C_2g_ 2	0.10	5.63	1.44
	C_2g_ 3	0.03	1.32	1.90
	C_2g_ 4	0.17	9.76	1.46
	C_2g_ 5	0.10	5.19	1.41
	C_2g_ 6	0.07	3.69	1.29
	C_2g_ 7	0.17	8.87	1.21
	C_2g_ 8	0.17	9.76	1.46
	C_2g_ 9	0.10	5.19	1.41
RBSHA3	C_3g_ 1	0.18	8.36	2.06
	C_3g_ 2	0.19	9.49	1.37
	C_3g_ 3	0.04	2.06	1.66
	C_3g_ 4	0.20	9.35	1.52
	C_3g_ 5	0.07	3.22	1.81
	C_3g_ 6	0.17	8.22	1.43
	C_3g_ 7	0.11	4.63	2.02
	C_3g_ 8	0.04	2.06	1.66
**Strains**	**HA titer range (mg/ml)**			
RBSHA	0.08–0.55			
RBSHA2	1.21–1.90			
RBSHA3	1.37–2.06			

## Discussion

Microbial fermentation has become the main and ideal platform for industrial production of HA in recent years (Rodriguez-Marquez et al. 2022). Besides the natural producers, heterologous hosts are consolidated platforms for the production of HA (Manfrão-Netto et al. 2022). *B*. *subtilis* as an amenable organism for genetic manipulation, low demanding, safe and robust cell factory can be an ideal heterologous host strain. Accordingly, here we successfully developed recombinant *B*. *subtilis* strains for HA production including RBSHA, RBSHA2, and RBSHA3. The results showed that all developed strains are able to produce low molecular weight hyaluronic acid (< 30 kDa) with a narrow low polydispersity index (PDI < 0.5) in a completely reproducible mode. This is a significant achievement as previous studies have encountered difficulties in producing low molecular weight HA with low PDI.

Statistical analysis of HA titration among recombinant strains in three discrete production runs indicates that there is a significant difference (p-value < 0.05) between RBSHA2 and RBSHA3 in comparison with RBSHA strains. The highest HA titer belonged to one of the RBSHA3 clones (2.06 mg/ml), in which the HA titer is among the highest previously reported HA production by *E. coli* or *B. subtilis* strains harboring *sehas*A gene (Manfrão-Netto et al. 2022). The same results were obtained with another recombinant *B. subtilis*, RBSHA2 strain harboring integrated *has*A along with replicative forms of *tua*D/*gta*B genes (1.90 mg/ml). However, Statistical analysis indicates that there is no significant difference (p-value > 0.05) in HA production between RBSHA2 and RBSHA3 strains. Regarding to the fact that both HA biosynthesis precursors are required for cell wall biosynthesis, and also the level of UDP-GlcUA is limited in *B*. *subtilis* due to insufficient levels of UDP-Glc dehydrogenase enzyme (Westbrook et al. 2018a). Therefore, it is expected that overexpression of endogenous UDP-GlcUA biosynthetic pathway genes, in particular *tua*D and *gta*B genes, would improve the HA production. As our results showed, RBSHA2 produced HA titer about four-fold higher than that of RBSHA strains. Winder et al. reported multi-grams per liter (the exact amount is not reported) of HA for *B. subtilis* carrying the integrated *has*A, *tua*D, and *gta*B genes in the presence of sucrose. This carbon source provided glucose and fructose as primary substrates for both HA precursors in the biosynthesis pathway (Widner et al. 2005). In another similar study, Chien et al. reported the production of 1.8 mg/ml HA by recombinant *B*. *subtilis* strain harboring the integrated *has*A/*tua*D/*vgb* genes at the end of cultivation (Chien and Lee 2007). The higher titer of HA production in our study compared to mentioned experiments can be related to the replicative expression of *tua*D/*gta*B genes that leads to intracellular availability of precursors and therefore increase of HA titer.

It was indicated that insufficient oxygen supply due to the increased viscosity of the culture broth in HA-producing strains can restrict HA production. This phenomenon was explained by the low dissolved oxygen available in the culture medium (Manfrão-Netto et al. 2022). In contrast to the previous study (Leroux et al. 2007) that found co-expression of a bacterial hemoglobin (VHb) would enhance the HA titer due to improvement of cell growth and energy flow, our results did not show any significant effect on the HA production by RBSHA3 strain. In justifying this phenomenon, it should be noted that the effect of *Vitreoscilla* hemoglobin (VHb) on the production of HA varies depending on the specific strain and the gene construct design strategy employed. While VHb expression was predicted to increase ATP levels and improve oxygen transfer and cell intake, the results of many studies in this field were not always consistent and contrary results were reported in different strains. In some cases, VHb expression had a negative impact on HA synthesis, while in other cases, it enhanced HA production. For example, in *Corynebacterium glutamicum*, co-expression of the VHb gene (*vgb*) with *has*A lowered HA yield by about 1.5-fold and had a negative impact on HA synthesis (Hoffmann and Altenbuchner 2014; Manfrão‐Netto et al. 2022). However, in another study in engineered *C*. *glutamicum*, genome-integrated strategy was employed to express VHb for oxygen transfer and cell intake enhancements. They reported that after introduction of VHb, intracellular ATP and NAD + increased dramatically, and they supposed that the integration of the VHb gene into the host genome is a promising strategy for industrialized HA bioproduction(Du et al. 2021). Similarly, in the study conducted by Leroux et al. co-expression of a bacterial hemoglobin (VHb) was found to enhance HA titer, and they also employed an integrated *vgb* gene(Leroux et al. 2007). The effects of VHb on ATP levels are also not consistent, with reported effects ranging from increases, decreases, or no change(Webster et al. 2021). In our study, the aim was to investigate whether the growth and productivity advantages seen in *E*. *coli* expressing VHb could be extended to our host using the replicative gene construct design strategy employed. Given that VHb can improve respiration and energy metabolism under oxygen-limited condition(Zhao et al. 2017), the lack of a remarkable effect of VHb expression in our experiment might be due to the mini-scale of HA production and/or low molecular weight of produced HA, which probably cannot lead to an oxygen restriction, which is a remarkable trigger for the manifestation of VHb effect. It is also important to note that VHb can affect the transcriptional levels of hundreds of genes, representing a variety of biochemical pathways(Webster et al. 2021). The VHb expression may affect the levels of many compounds of intermediary metabolism and apparently alter the expression of many genes. Thus, the metabolic changes in organisms engineered to express VHb are likely to be numerous and complicated(Stark et al. 2012). This complexity makes it more difficult to identify the factors affecting the performance of VHb and the resulting effects on HA production. In summary, while the effect of VHb on HA production can vary depending on the specific strain and gene construct design strategy employed, it is clear that VHb has a complex and multifaceted impact on cellular metabolism.

Based on the amylase test, although multimeric forms had a higher transformation rate, but they did not show significant effect on HA production in all recombinant strains (p-value > 0.05). The control of Mw and size distribution of HA is a multifactorial mechanism that depends on HAS enzyme structure, host background, micro-environment, substrate concentration, and culture conditions. The Mw of HA is thought to depend on the availability of the precursors, both of which are suggested by some studies to be required in nearly equimolar concentrations (Manfrão-Netto et al. 2022). On the other hand, in the presence of excess UDP-GlcUA or UDP-GlcNAc, chain elongation might stall temporarily but the chain is retained until the appropriate substrate is available for synthesis and chain elongation to commence. As a result, in our experiment, probably the lack of sufficient and available UDP-GlcNAc substrate can explain the short length of the produced HA chain. Because it has shown that the balance between intracellular substrate levels of UDP-GlcUA and UDP-GlcNAc is necessary for the high-level production of high Mw HA in engineered *B*. *subtilis* (Westbrook et al. 2018b). A wide range of Mw for HA has been reported by recombinant *B. subtilis* (from 2.2 to 6973 kDa) (Manfrão‐Netto et al. 2022). HA with Mw range of 20–200 kDa mediates biological functions such as embryonic development, wound healing, and ovulation. This fact that the HA Mw dictates its functions highlights the production of low Mw and monodisperse HA for medical applications (Snetkov et al. 2020).

The Mw analysis by gel electrophoresis demonstrated that all our recombinant strains produced the same low Mw of HA 30 kDa (Online Resource Fig. 7 and Fig. 10). Moreover, the produced HAs were relatively monodisperse as measured by DLS technique. Since the polydispersity index (PDI) is an important parameter that reflects the heterogeneity of the molecular weight distribution of HA, our study stands out from previous works as we achieved a significantly lower PDI compared to other studies that have produced HA with high molecular weight. For example, Winder et al. reported a PDI of 1.5 with a high molecular weight of 1.15 MDa(Widner et al. 2005). Cerminati and Chien did not investigate polydispersity, and Jia et al. reported a PDI between 1.13 and 1.68 in all their five recombinant strains, which is significantly higher than the PDI achieved in our study(Cerminati 2021; Chien and Lee 2007; Jia et al. 2013). Jin has succeeded in producing hyaluronic acid with low molecular weight but by using co-expression of leech hyaluronidase which in turn can lead to the degradation of the produced hyaluronic acid chain and potentially increase polydispersity which requires further genetic engineering strategies to control leech hyaluronidase activity; the PDI reported for 13 recombinant strains was ranged from 1.06 to 1.26 as well(Jin et al. 2016). Westbrook also reported to achieve high molecular weight hyaluronic acid and also did not report any data on polydispersity of produced biopolymer(Westbrook et al. 2018b). Li et al. has also reported the production of hyaluronic acid with molecular weight ranging from 0.392 to 6.937 MDa, and there is no reports regarding the polydispersity of produced hyaluronic acid (Li et al. 2019).

Furthermore, our study employed an excellent strategy of two different gene constructs designed in the form of an integrated *has*A gene in the chromosome along with overexpression of precursor genes in the form of extrachromosomal plasmids. This approach allowed us to achieve low molecular weight HA with low PDI in a reproducible manner. Given the fact that low molecular weight HA is desirable for certain medical applications such as wound healing and ovulation(Snetkov et al. 2020), our study represents a significant achievement in the field of HA production for medical applications.

In the case of HA production by bacterial fermentation, the most important factors are production rate, control of Mw, low polydispersity index and optimized purification procedures. It should be noted that HA production in recombinant microorganisms was mostly polydisperse, a characteristic that limits its commercial value and applications (Boeriu et al. 2013; de Oliveira et al. 2016). In the present study, three recombinant *B. subtilis* stains were successfully developed with ability to low Mw and nearly monodisperse HA production. Certainly, combinational manipulation of the central metabolic pathway of HA biosynthesis and also competitive pathways will provide sufficient precursors, cofactors, and energy require for HA-producing hosts.

## Electronic supplementary material

Below is the link to the electronic supplementary material.

Supplementary Material 1

## Data Availability

The data used to support the results of this study is embedded within this article and its supporting file as “online resource”.

## Abbreviations

ATP: Adenosine triphosphate
*amy*E: Amylase gene of *Bacillus subtilis*
*B.* subtilis: *Bacillus subtilis*
DO: Dissolved oxygen
FTIR: Fourier-transform infrared spectroscopy
GlcUA: Glucuronic acid
GlcNAc: N-acetyl glucoseamine
GRAS: Generally recognized as safe
*gta*B: *Bacillus subtilis* UDP-Glucose pyrophosphorylase gene
h: hour
HA: Hyaluronic acid
HAS: Hyaluronan synthase enzyme
*has*A: *Streptococcus* Hyaluronan synthase gene
*has*B: *Streptococcus* UDP-Glucose dehydrogenase gene
*has*C: *Streptococcus* UDP-Glucose pyrophosphorylase gene
IPTG: Isopropyl β-D-1-thiogalactopyranoside
kDa: kilo Dalton
Mw: Molecular weight
mopG2: Monomer form of pG2 plasmid
mupG2: Multimer form of pG2 plasmid
mopG3: Monomer form of pG3 plasmid
mupG3: Multimer form of pG3 plasmid
mopDHhas: Monomer form of pDHhas plasmid
mupDHhas: Multimer form of pDHhas plasmid
OD: Optical density
seHAS: *Streptococcus equisimilis* Hyaluronan synthase enzyme
*S. equisimilis*: *Streptococcus equisimilis*
TCA: Trichloroacetic acid
*tua*D: *Bacillus subtilis* UDP-Glucose dehydrogenase gene
TuaD: *Bacillus subtilis* UDP-Glucose dehydrogenase enzyme
*gta*B: *Bacillus subtilis* UDP-Glucose pyrophosphorylase gene
GtaB: *Bacillus subtilis* UDP-Glucose pyrophosphorylase enzyme
UDP: Uridine diphosphate
*vgb*: *Vitreoscilla* hemoglobin gene
VHb: *Vitreoscilla* hemoglobin.

## References

[CR1] Amjad Zanjani FS, Afrasiabi S, Norouzian D, Ahmadian G, Hosseinzadeh SA, Fayazi Barjin A, Cohan RA, Keramati M (2022). Hyaluronic acid production and characterization by novel *Bacillus subtilis* harboring truncated Hyaluronan synthase. AMB Express.

[CR2] Anagnostopoulos C, Spizizen J (1961). Requirements for transformation in *Bacillus subtilis*. J Bacteriol.

[CR3] Boeriu CG, Springer J, Kooy FK, van den Broek LA, Eggink G (2013) Production methods for hyaluronan. International Journal of Carbohydrate Chemistry 2013

[CR4] Bowman S, Awad ME, Hamrick MW, Hunter M, Fulzele S (2018). Recent advances in hyaluronic acid based therapy for osteoarthritis. Clin translational Med.

[CR5] Bukhari SNA, Roswandi NL, Waqas M, Habib H, Hussain F, Khan S, Sohail M, Ramli NA, Thu HE, Hussain Z (2018). Hyaluronic acid, a promising skin rejuvenating biomedicine: a review of recent updates and pre-clinical and clinical investigations on cosmetic and nutricosmetic effects. Int J Biol Macromol.

[CR6] Cerminati S (2021). Low cost and sustainable hyaluronic acid production in a manufacturing platform based on *Bacillus subtilis* 3NA strain. 3 Biotech.

[CR7] Cesaretti M, Luppi E, Maccari F, Volpi N (2003). A 96-well assay for uronic acid carbazole reaction. Carbohydr Polym.

[CR8] Chen H, Qin J, Hu Y (2019). Efficient degradation of high-molecular-weight hyaluronic acid by a combination of ultrasound, hydrogen peroxide, and copper ion. Molecules.

[CR9] Chien LJ, Lee CK (2007). Enhanced Hyaluronic Acid production in *Bacillus subtilis* by coexpressing bacterial hemoglobin. Biotechnol Prog.

[CR10] de Oliveira JD, Carvalho LS, Gomes AMV, Queiroz LR, Magalhães BS, Parachin NS (2016). Genetic basis for hyper production of hyaluronic acid in natural and engineered microorganisms. Microb Cell Fact.

[CR11] Du Y, Cheng F, Wang M, Xu C, Yu H (2021) Indirect pathway metabolic engineering strategies for enhanced biosynthesis of hyaluronic acid in engineered Corynebacterium glutamicum. Frontiers in Bioengineering and Biotechnology:1273 10.3389/fbioe.2021.768490 PMC8721151 34988066

[CR12] Gilli R, Kacuráková M, Mathlouthi M, Navarini L, Paoletti S (1994). FTIR studies of sodium hyaluronate and its oligomers in the amorphous solid phase and in aqueous solution. Carbohydr Res.

[CR13] Green M, Sambrook J, Sambrook J (2012). Molecular cloning: a laboratory manual 4 edition Cold.

[CR14] Gunasekaran V, Gowdhaman D, Ponnusami V (2020) Role of membrane proteins in bacterial synthesis of hyaluronic acid and their potential in industrial production. International Journal of Biological Macromolecules 10.1016/j.ijbiomac.2020.08.077 32791275

[CR15] Harwood CR (1992). Bacillus subtilis and its relatives: molecular biological and industrial workhorses. Trends Biotechnol.

[CR16] Hoffmann J, Altenbuchner J (2014). Hyaluronic acid production with Corynebacterium glutamicum: effect of media composition on yield and molecular weight. J Appl Microbiol.

[CR17] Jia Y, Zhu J, Chen X, Tang D, Su D, Yao W, Gao X (2013). Metabolic engineering of *Bacillus subtilis* for the efficient biosynthesis of uniform hyaluronic acid with controlled molecular weights. Bioresour Technol.

[CR18] Jin P, Kang Z, Yuan P, Du G, Chen J (2016). Production of specific-molecular-weight hyaluronan by metabolically engineered *Bacillus subtilis* 168. Metab Eng.

[CR19] Lal A, Cheeptham N (2012) Starch agar protocol. Am Soc Microbiol :1–9

[CR20] Leroux M, Anselmi P, Peirú S, Alonso JC, Priem B, Menzella HG, Chien LJ, Lee CK (2007). Enhanced hyaluronic acid production in *Bacillus subtilis* by coexpressing bacterial hemoglobin. Appl Microbiol Biotechnol.

[CR21] Li Y, Li G, Zhao X, Shao Y, Wu M, Ma T (2019) Regulation of hyaluronic acid molecular weight and titer by temperature in engineered *Bacillus subtilis*. 9(6):225. 10.1007/s13205-019-1749-x 10.1007/s13205-019-1749-x PMC6529495 31139540

[CR22] Liu L, Liu Y, Li J, Du G, Chen J (2011). Microbial production of hyaluronic acid: current state, challenges, and perspectives. Microb Cell Fact.

[CR23] Manfrão-Netto JH, Queiroz EB, de Oliveira Junqueira AC, Gomes AM, Gusmao de Morais D, Paes HC, Parachin NS (2022). Genetic strategies for improving hyaluronic acid production in recombinant bacterial culture. J Appl Microbiol.

[CR24] Radrezza S, Baron G, Nukala SB, Depta G, Aldini G, Carini M, D’Amato A (2020). Advanced quantitative proteomics to evaluate molecular effects of low-molecular-weight hyaluronic acid in human dermal fibroblasts. J Pharm Biomed Anal.

[CR25] Rajalingam D, Loftis C, Xu JJ, Kumar TKS (2009). Trichloroacetic acid-induced protein precipitation involves the reversible association of a stable partially structured intermediate. Protein Sci.

[CR26] Rodriguez-Marquez CD, Arteaga-Marin S, Rivas-Sánchez A, Autrique-Hernández R, Castro-Muñoz R (2022). A review on current strategies for extraction and purification of Hyaluronic Acid. Int J Mol Sci.

[CR27] Schiraldi C, Andreozzi L, Marzaioli I, Vinciguerra S, D’Avino A, Volpe F, Panariello A, De Rosa M (2010). Hyaluronic acid degradation during initial steps of downstream processing. Biocatal Biotransform.

[CR28] Schiraldi C, La Gatta A, De Rosa M (2010b) Biotechnological production and application of hyaluronan. INTECH Open Access Publisher

[CR29] Snetkov P, Zakharova K, Morozkina S, Olekhnovich R, Uspenskaya M (2020). Hyaluronic acid: the influence of molecular weight on structural, physical, physico-chemical, and degradable properties of biopolymer. Polymers.

[CR30] Stark BC, Dikshit KL, Pagilla KR (2012). The biochemistry of Vitreoscilla hemoglobin. Comput Struct Biotechnol J.

[CR31] Stragier P, Bonamy C, Karmazyn-Campelli C (1988). Processing of a sporulation sigma factor in *Bacillus subtilis*: how morphological structure could control gene expression. Cell.

[CR32] Toymentseva AA, Schrecke K, Sharipova MR, Mascher T (2012). The LIKE system, a novel protein expression toolbox for *Bacillus subtilis* based on the liaI promoter. Microb Cell Fact.

[CR33] Ucm R, Aem M, Lhb Z, Kumar V, Taherzadeh MJ, Garlapati VK, Chandel AK (2022). Comprehensive review on biotechnological production of hyaluronic acid: status, innovation, market and applications. Bioengineered.

[CR34] Voß C, Schmidt T, Schleef M, Friehs K, Flaschel E (2003). Production of supercoiled multimeric plasmid DNA for biopharmaceutical application. J Biotechnol.

[CR35] Webster DA, Dikshit KL, Pagilla KR, Stark BC (2021). The discovery of Vitreoscilla hemoglobin and early studies on its biochemical functions, the control of its expression, and its use in practical applications. Microorganisms.

[CR36] Westbrook AW, Moo-Young M, Chou CP (2016). Development of a CRISPR-Cas9 tool kit for comprehensive engineering of *Bacillus subtilis*. Appl Environ Microbiol.

[CR37] Westbrook AW, Ren X, Moo-Young M, Chou CP (2018a) Engineering of cell membrane to enhance heterologous production of hyaluronic acid in *Bacillus subtilis*. 115(1):216–231. 10.1002/bit.26459 10.1002/bit.26459 28941282

[CR38] Westbrook AW, Ren X, Oh J, Moo-Young M, Chou CP (2018). Metabolic engineering to enhance heterologous production of hyaluronic acid in *Bacillus subtilis*. Metab Eng.

[CR39] Widner B, Behr R, Von Dollen S, Tang M, Heu T, Sloma A, Sternberg D, DeAngelis PL, Weigel PH, Brown S (2005). Hyaluronic acid production in *Bacillus subtilis*. Appl Environ Microbiol.

[CR40] Yamamoto H, Serizawa M, Thompson J, Sekiguchi J (2001). Regulation of the glv operon in *Bacillus subtilis*: YfiA (GlvR) is a positive regulator of the operon that is repressed through CcpA and cre. J Bacteriol.

[CR41] Zhao T-X, Li M, Zheng X, Wang C-H, Zhao H-X, Zhang C, Xing X-H (2017). Improved production of trans-4-hydroxy-l-proline by chromosomal integration of the Vitreoscilla hemoglobin gene into recombinant Escherichia coli with expression of proline-4-hydroxylase. J Biosci Bioeng.

