# Supplementary material for: The effect of manipulating glucuronic acid biosynthetic pathway in Bacillus subtilis strain on hyaluronic acid production
Source: AMB Express. 2023 Jun 24;13:63. doi: 10.1186/s13568-023-01567-2 (PMC10290625; doi:10.1186/s13568-023-01567-2)
Supplement: Supplementary file 1 — Supplementary Material 1 [file 13568_2023_1567_MOESM1_ESM.docx]

**The effect of manipulating glucuronic acid biosynthetic pathway in *Bacillus* *subtilis* strain on hyaluronic acid production**

Shadi Afrasiabi^1^, Fatemeh Sadat Amjad Zanjani^1^, Gholamreza Ahmadian^2^, Reza Ahangari Cohan^1*^, Malihe Keramati^1*^

^1^Department of Nanobiotechnology, New Technologies Research Group, Pasteur Institute of Iran, Tehran, Iran

^2^Department of Industrial and Environmental Biotechnology, National Institute for Genetic Engineering and Biotechnology (NIGEB), Tehran, Iran

***Corresponding authors:**

Department of Nanobiotechnology, New Technologies Research Group, Pasteur Institute of Iran, Tehran, Iran, Tel: (98)2164112126, Email: [cohan_r@yahoo.com](mailto:cohan_r@yahoo.com), and

[keramati.malihe@gmail.com](mailto:keramati.malihe@gmail.com), [keramatim@pasteur.ac.ir](mailto:keramatim@pasteur.ac.ir) , 0000-002-7196-8751

**Table 1.** Primers used in this study

| Primer Name | Sequence (5ˊ→ 3ˊ) |
| --- | --- |
| hasA-F | TCCAGAACAACCTCTGCTAAAATTCC |
| hasA-IF | TCTAATGTTATCGTTCATCGCTCAG |
| hasA-R | CGAGGTCATCATTTCCTTCCGAA |

**Table 2.** Nucleic acid and protein impurities in purified HA samples.

| **Strains** | DNA impurity | Protein impurity | |
| --- | --- | --- | --- |
|  | (A_260 nm_) | (mg/L) | % |
| RBSHA | 0/068±0/026 | 0.6±0.2 | 0.06±0.2 |
| RBSHA2 | 0/030±0/004 | 0.4±0.3 | 0.04±0.3 |
| RBSHA3 | 0/03±0/007 | 0.3±0.3 | 0.03±0.3 |
| Acceptance limit according to EP (HA monograph) ^a^ | ≤0.5 | ≤1 | ≤0.1 |


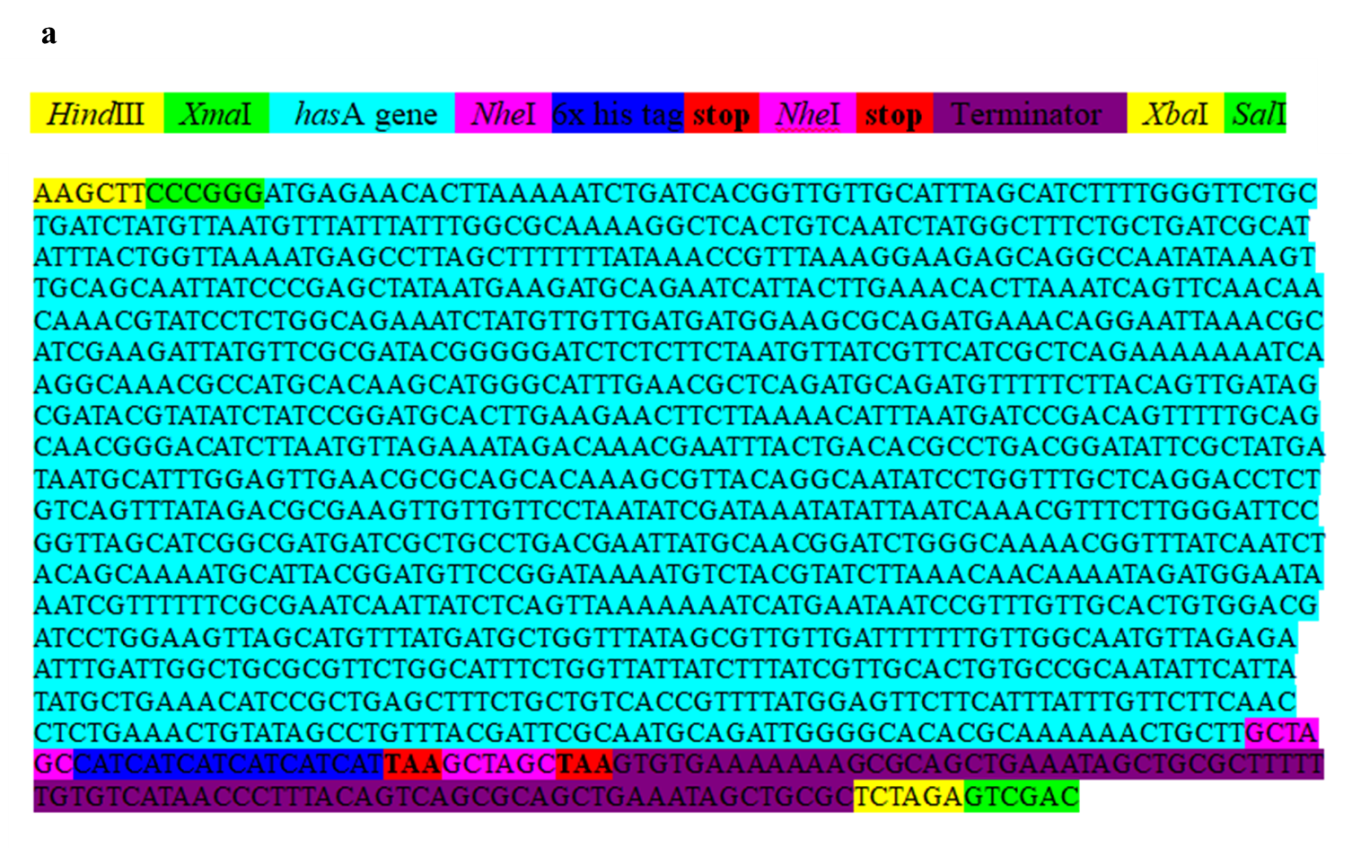


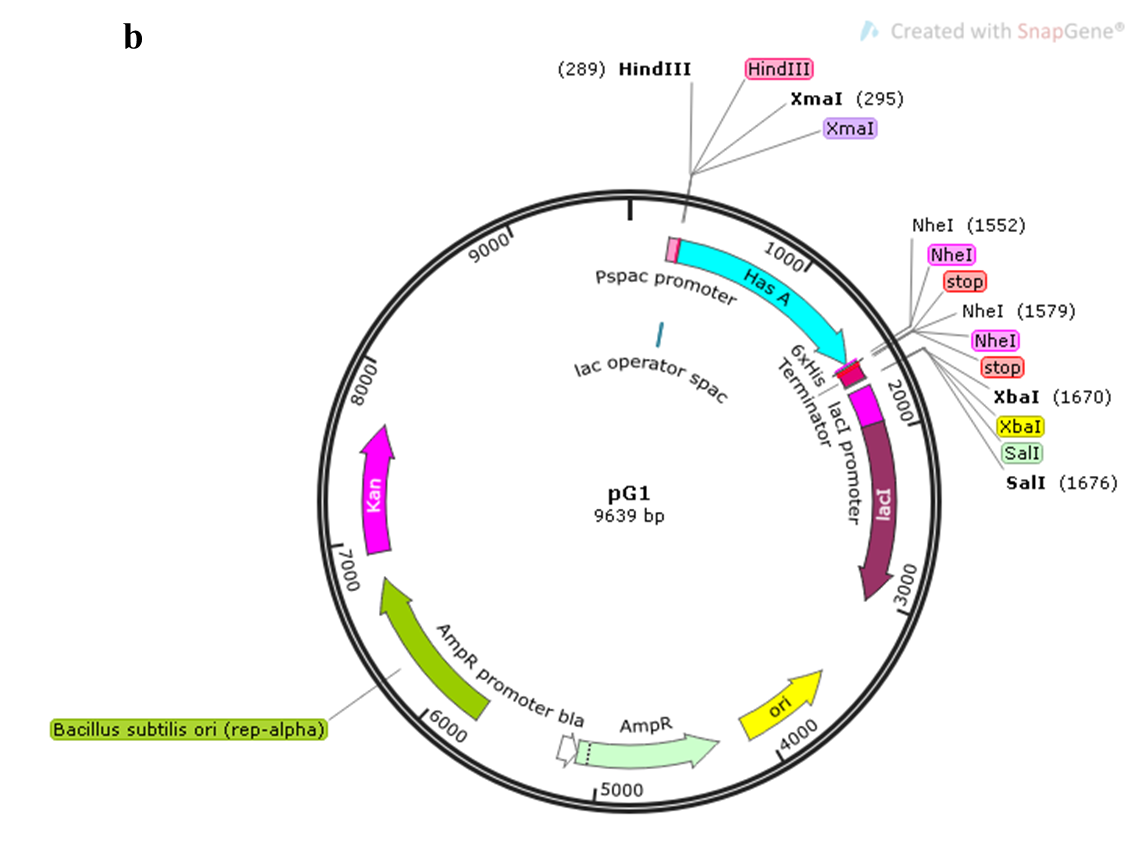


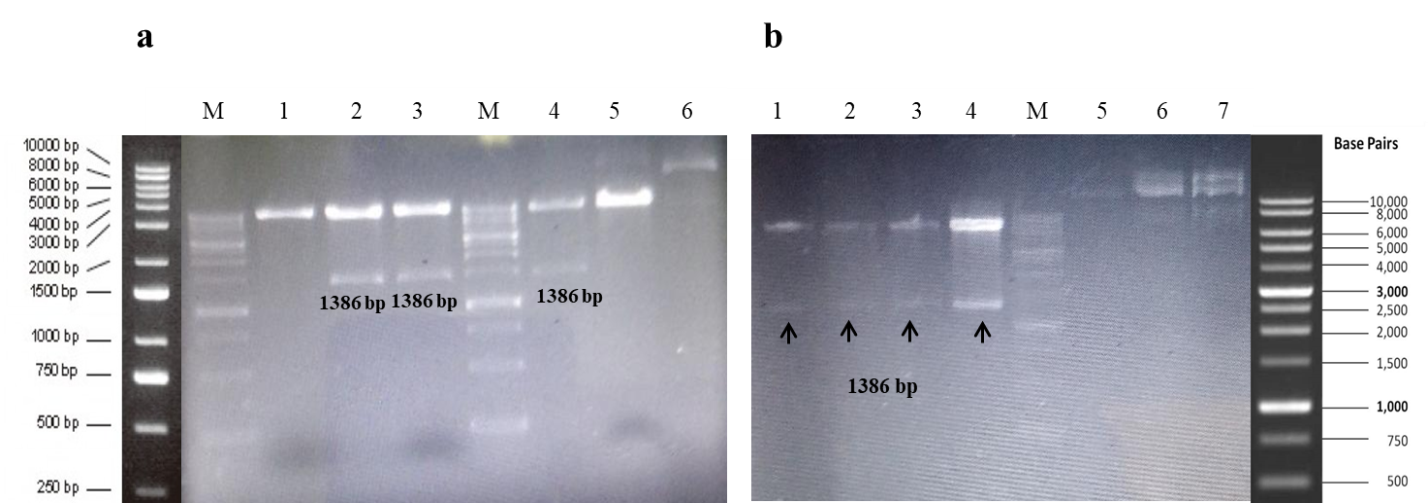


**C**

**Fig.1 Integrative expression construct.** **(a)** Integrative construct sequence harboring 1251bp *has*A gene along with restriction sites, stop codons, and terminator sequence. **(b)** Schematic representation of recombinant *E. coli-B. subtilis* shuttle expression vector pG1 including *has*A gene. **(c) Agarose gel electrophoresis of *Hin*dIII-*Xba*I digested pDHhas: (a)** Agarose gel electrophoresis of *Hin*dIII-*Xba*I digested mopDHhas [M: DNA marker; lanes 1 to 5: *Hin*dIII-*Xba*I digested mopDHhas from five *E.coli* Top10 recombinants*;* lane 6: undigested mopDHhas]. A 1386bp fragment of *has*A gene in examined clones is shown in lanes 2, 3 and 4. **(b)** Agarose gel electrophoresis of *Hin*dIII-*Xba*I digested mupDHhas [Lanes 1 to 4: *Hin*dIII*-Xba*I digested mupDHhas*;* M: DNA marker; lane 5: pDHAFB backbone; lanes 6 and 7: undigested mupDHhas]. A 1386bp fragment of *has*A gene in examined clones is shown in lanes 1, 2, 3, and 4.


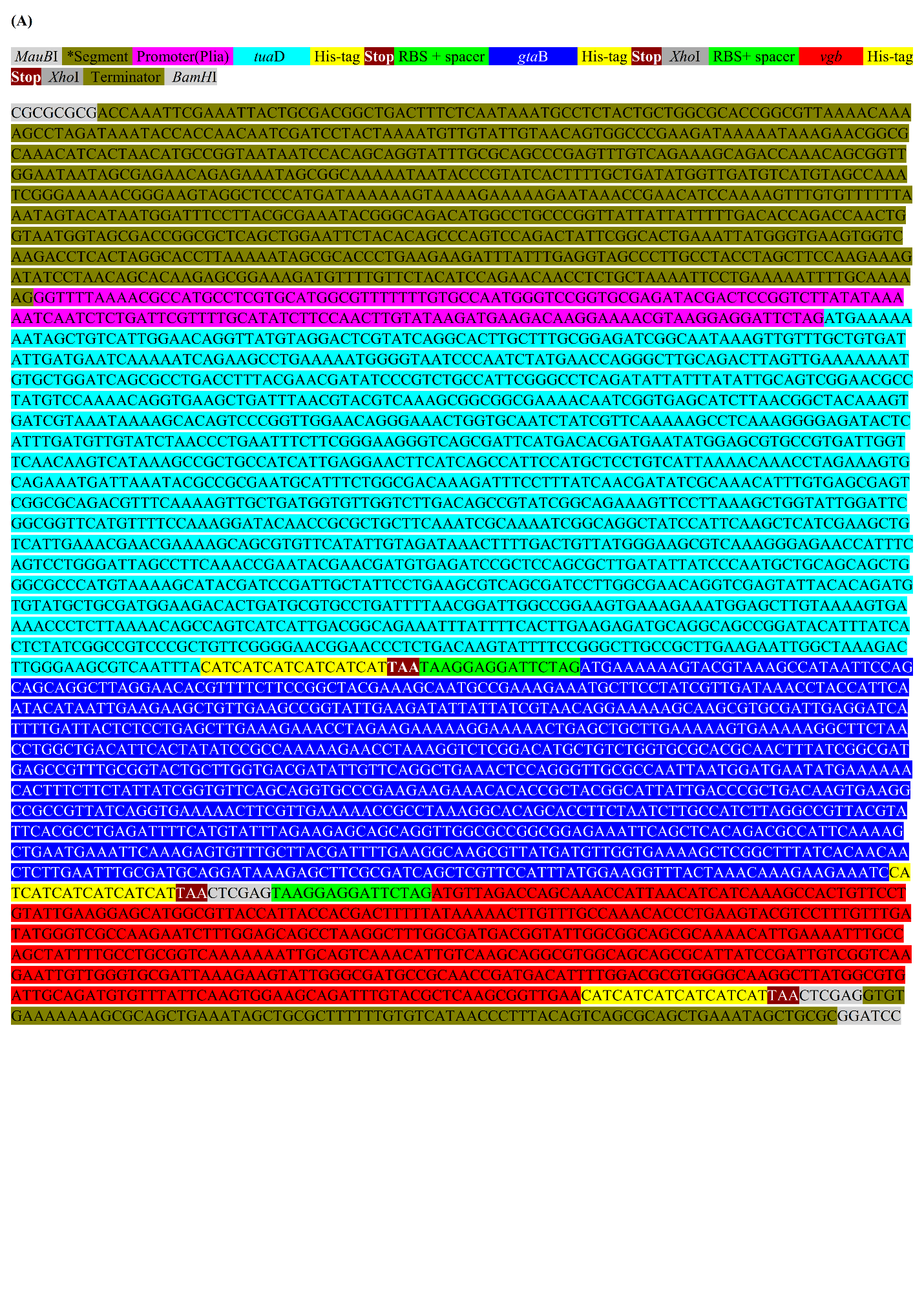


**aC**


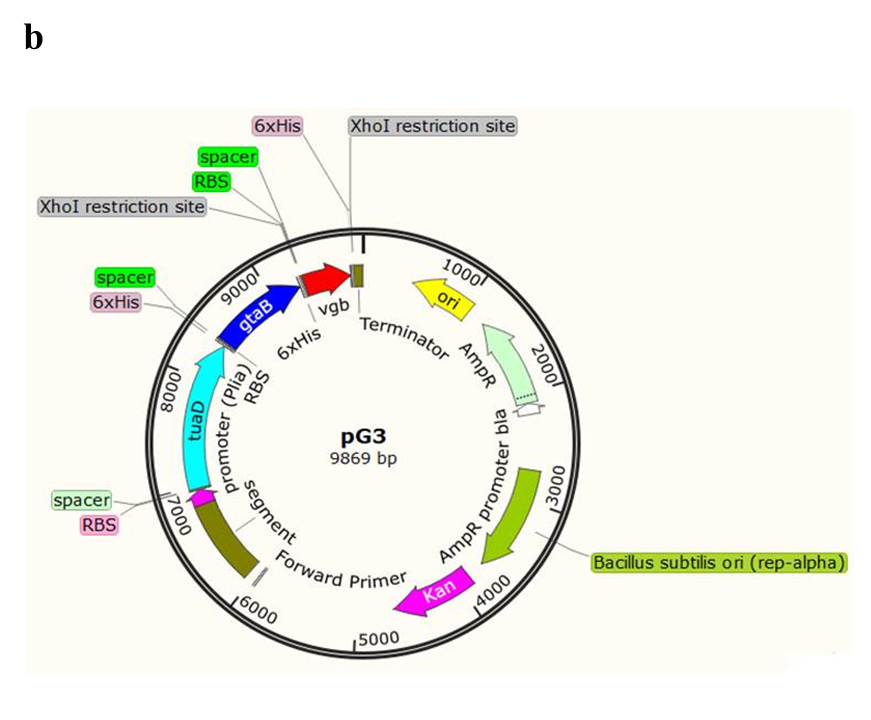


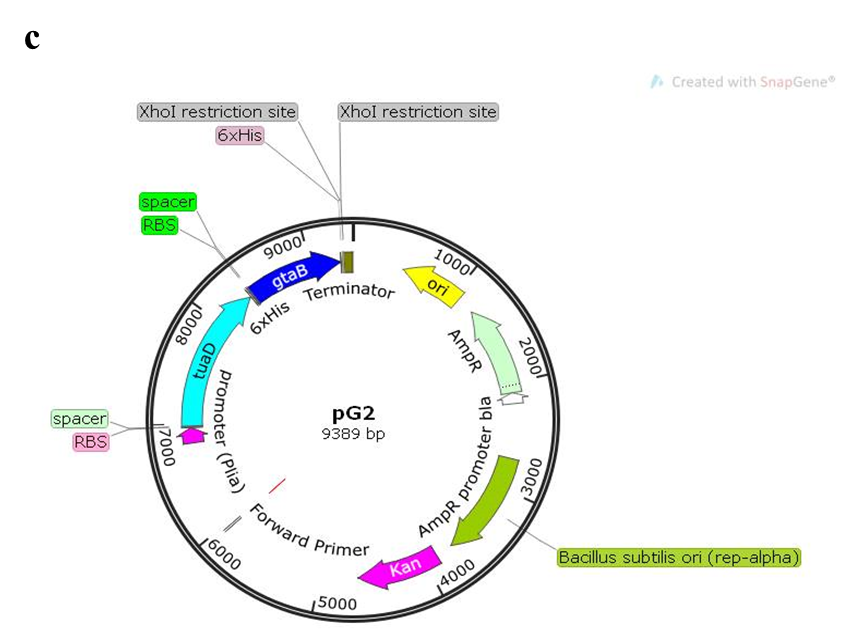


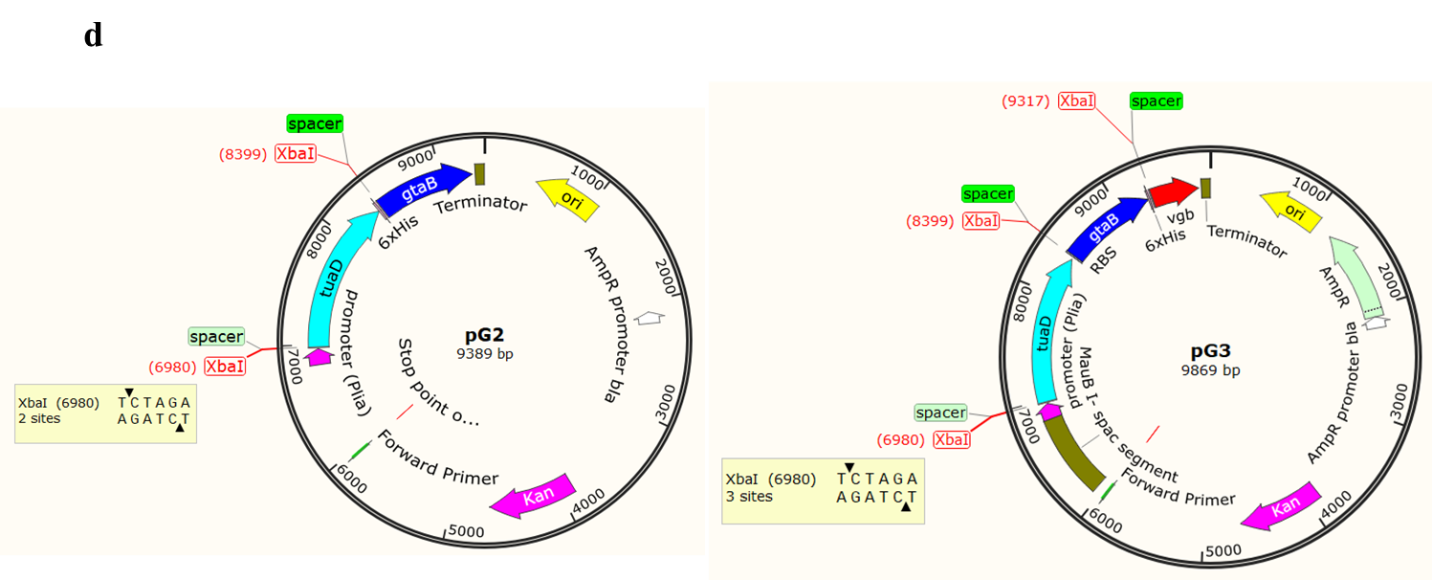


**Fig.2 Replicative expression construct.** **(a)** Replicative construct sequence harboring *tua*D/*gta*B*/vgb* genes along with restriction sites, stop codons, and terminator sequence. **(b)** Schematic representation of recombinant *E. coli-B. subtili*s shuttle expression vector pG3. **(c)** Schematic representation of recombinant *E. coli-B. subtili*s shuttle expression vector pG2. **(d)** *Xba*I recognition sites on recombinant *E. coli-B. subtili*s shuttle expression vector pG2 and pG3.


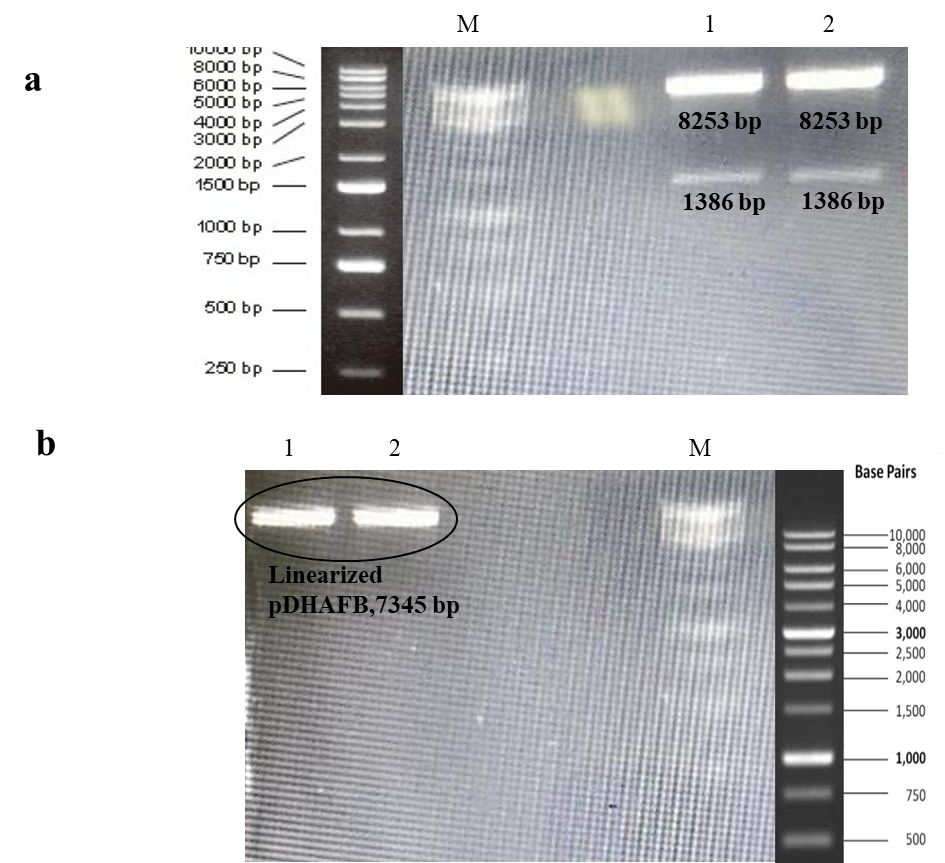


**Fig.3 Agarose gel electrophoresis of *Hin*dIII-*Xba*I double digested pG1 and pDHAFB plasmids. (a)** *Hin*dIII -*Xba*I double digested pG1. [M: DNA marker; lanes 1 and 2: *Hin*dIII -*Xba*I double digested pG1]. The *has*A gene fragment (1386 bp) and the linearized pDG148 plasmid backbone (8253 bp) are shown in lanes 1 and 2. **(b)** *Hin*dIII -*Xba*I double digested pDHAFB plasmid backbone. [M: DNA marker; lanes 1 and 2: *Hin*dIII-*Xba*I double digested pDHAFB]**.** The linearized pDHAFB band (7345 bp) flanked by *Hin*dIII and *Xba*I recognition sites is shown in lanes 1 and 2.

**
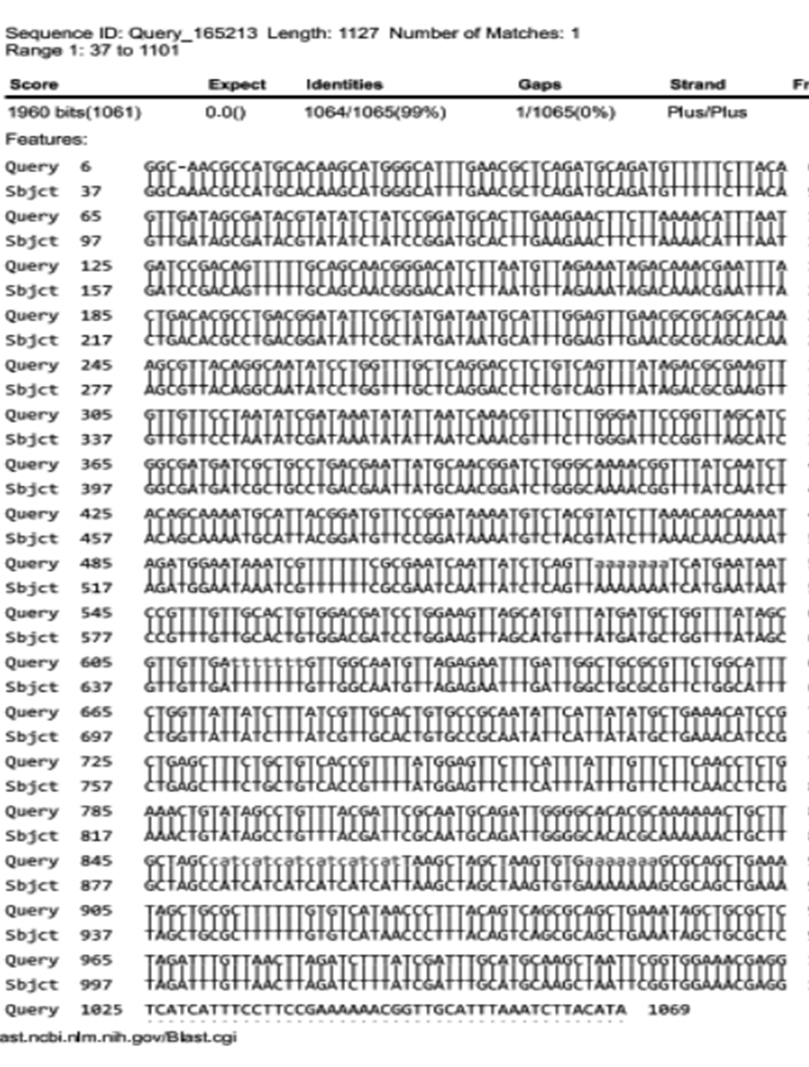
**

**Fig.4 Sequenced *has*A gene pairwise alignment result.** Pairwise alignment of sequenced *has*A gene.


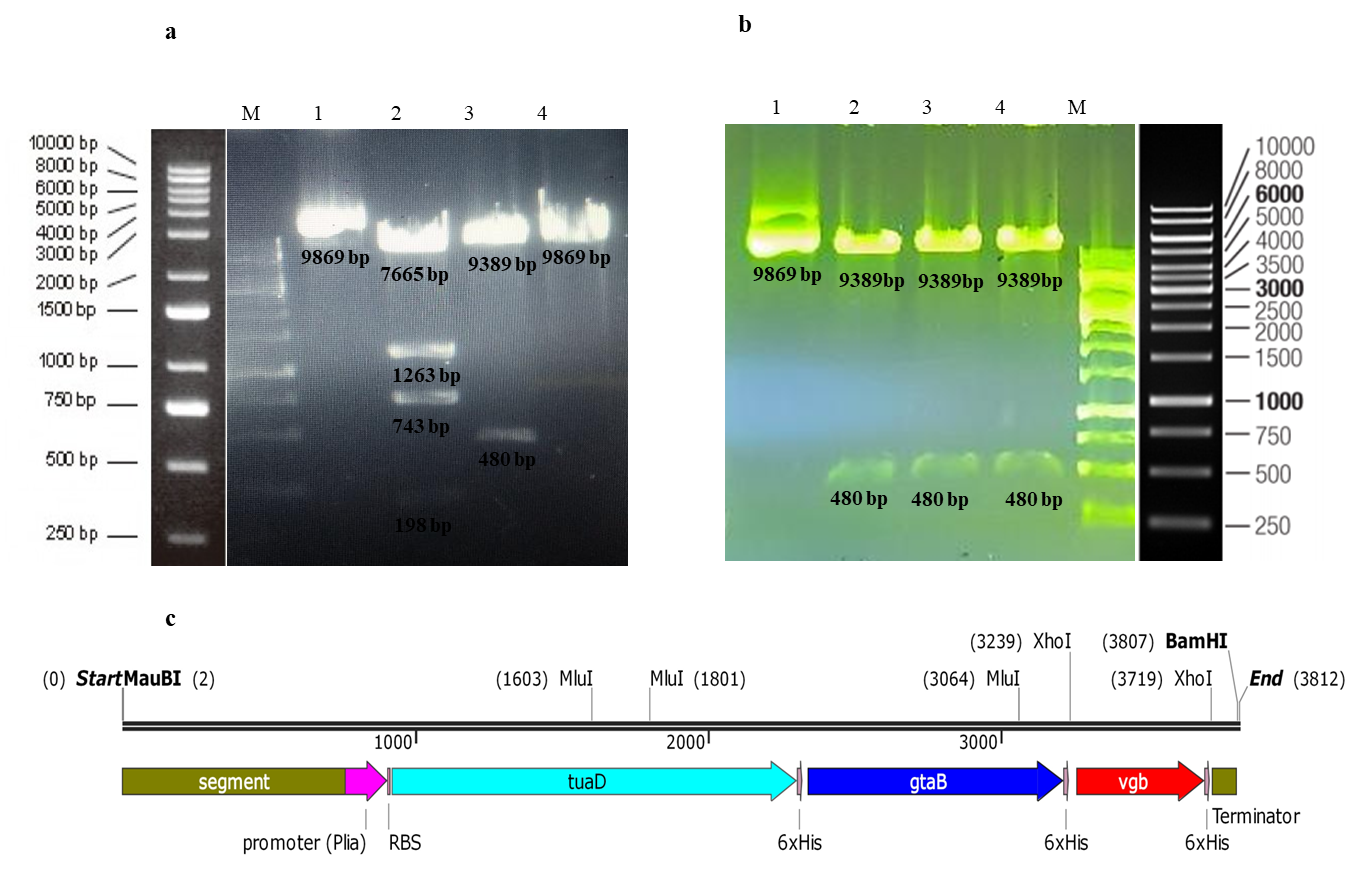
**Fig.5 Agarose gel electrophoresis of digested pG3 plasmid**. **(a)** Agarose gel electrophoresis of digested mopG3. [M: 1kb DNA size marker; lane 1: undigested mopG3; lane 2: *Mlu*I-*Bam*HI digested mopG3 including four fragments (7665, 1263, 743, and 198 bp); lane 3: *Xho*I digested mopG3 including two fragments (9389 and 480 bp), and lane 4: *Bam*HI digested mopG3 that shows a single band at 9869 bp from *E. coli* Top10 recombinant clone]. **(b)** Agarose gel electrophoresis of *Xho*I digested mupG3. [Lane1: undigested mupG3; lanes 2, 3, and 4: *Xho*I digested mupG3 from three *E.coli* C600 recombinant clones that each shows two fragments at 9389 and 480 bp; M: 1kb DNA size marker]. **(c)** Schematic representation of replicative construct in pG3 plasmid with restriction enzymes used for restriction map analysis.


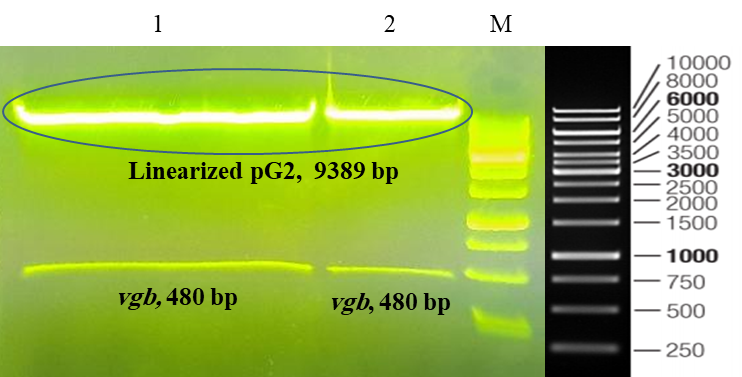


**Fig.6 Agarose gel electrophoresis of *Xho*I digested pG3 plasmid**. Lanes 1 and 2: *Xho*I digested pG3 including linearized pG2 at 9389 bp and *vgb* gene fragment at 480 bp; M: 1kb DNA size marker.


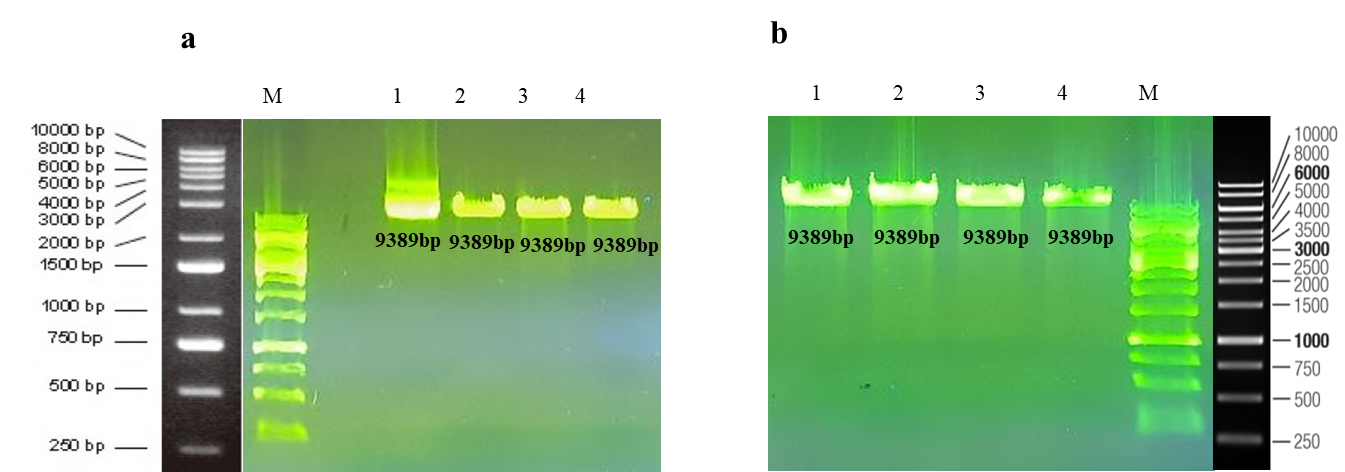


**Fig.7 Agarose gel electrophoresis of *Xho*I digested pG2 plasmid**. **(a)** Agarose gel electrophoresis of *Xho*I digested mupG2 plasmid [M: 1kb DNA size marker; lane 1: undigested mupG2; lanes 2 to 4: *Xho*I digested mupG2 from three recombinant *E.coli* C600 clones]. Linearized mupG2 (9389 bp) is shown in lanes 2 to 4. **(b)** Agarose gel electrophoresis of *Xho*I digested mopG2 plasmid*.* [Lanes 1 to 4: *Xho*I digested mopG2 from four recombinant *E.coli* Top10 clones; M: 1kb DNA size marker]. Linearized mopG2 (9389 bp) is shown in lanes 1 to 4.


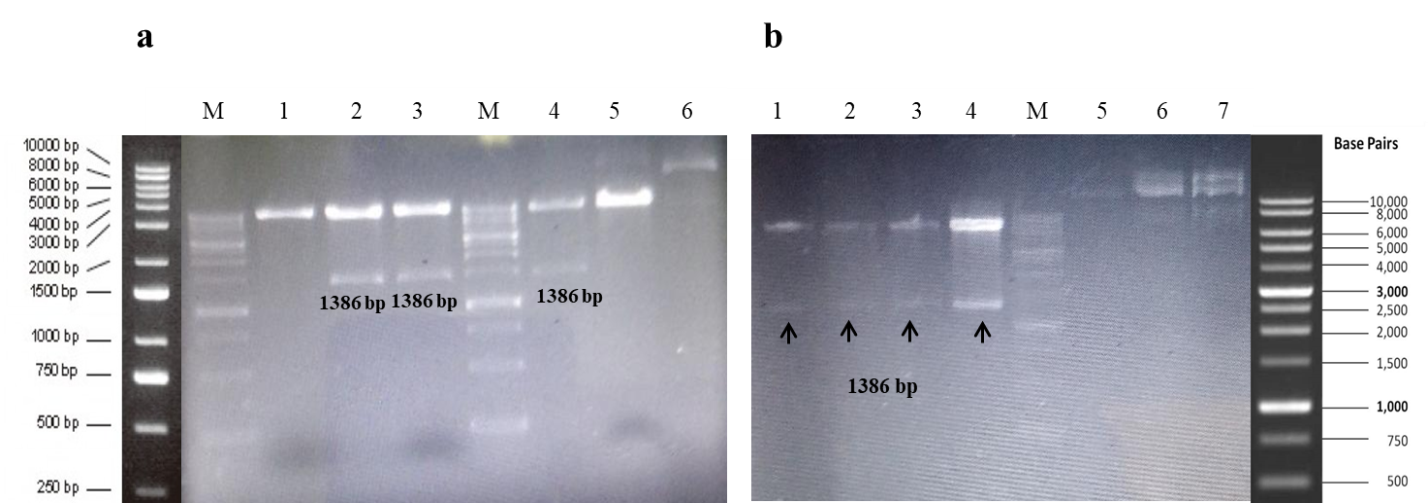


**Fig.8 Agarose gel electrophoresis of *Xba*I digested pG2 and pG3 extracted from RBSHA2 and RBSHA3 strains.** Lane 1: undigested pG2; lane 2: *Xba*I digested pG2; M: DNA marker; lane 3: undigested pG3; lane 4: *Xba*I digested pG3. Two fragments at 7970 and 1419 bp for the pG2 digestion and three bands at 7532, 1419, and 918 bp for the pG3 digestion were seen in lanes 2 and 4, respectively.


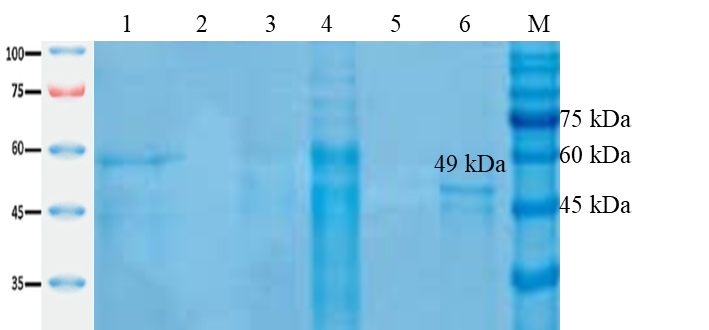


**Fig.9 SDS-PAGE analysis of recombinant HAS enzyme in IPTG-induced RBSHA strain.** Lanes 1, 2, and 3 are the wash, elution 1, and elution 2 of non-induced samples (control), respectively. Lanes 4, 5, and 6 are the wash, elution 1, and elution 2 of the induced sample, respectively. M is the protein marker. The corresponding band for recombinant HAS enzyme is seen at 49 kDa.


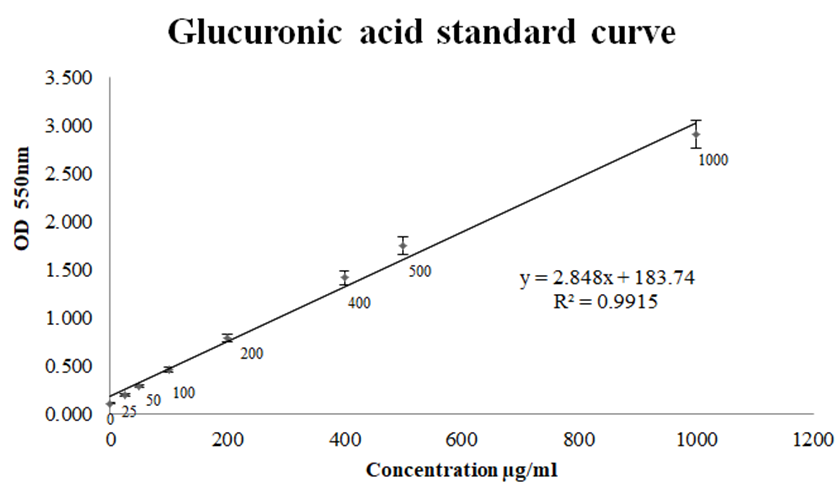


**Fig.10 Glucuronic acid standard curve**

**
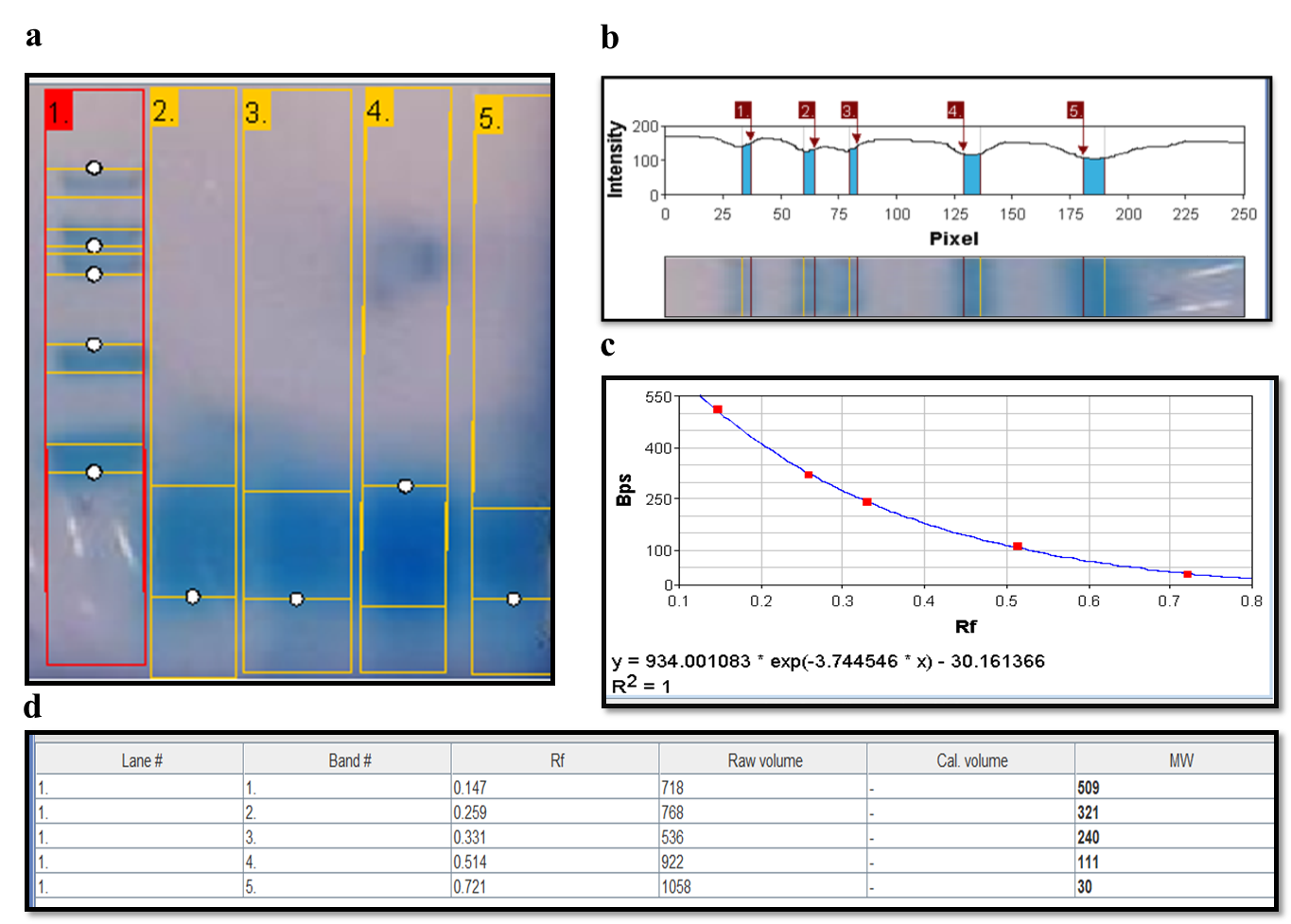
**

**Fig.11 The data of GelAnalyzer software for determination of HA Mw**. Briefly, the distance of relative migration (Rf) of HA bands in the ladder were measured (**a, b,** and **c**). Then, the known Mw of HA ladder (y-axis) were plotted against their relative migration (x-axis). A coefficient of determination (R2) of 1 was obtained from linear equation. **(d)** Rf and Mw of HA ladder were shown in the table.
